# CREPT/RPRD1B associates with Aurora B to regulate Cyclin B1 expression for accelerating the G2/M transition in gastric cancer

**DOI:** 10.1038/s41419-018-1211-8

**Published:** 2018-12-05

**Authors:** Lidan Ding, Liu Yang, Yuqi He, Bingtao Zhu, Fangli Ren, Xuanzi Fan, Yinyin Wang, Mengdi Li, Jun Li, Yanshen Kuang, Sihan Liu, Wanli Zhai, Danhui Ma, Yanfang Ju, Quentin Liu, Baoqing Jia, Jianqiu Sheng, Zhijie Chang

**Affiliations:** 1 State Key Laboratory of Membrane Biology, School of Medicine, Tsinghua University, Beijing, 100084 China; 2 Department of Gastroenterology, PLA Army General Hospital, Beijing, 100700 China; 3 Institute of Immunology, PLA, The Third Military Medical University, Chongqing, 400038 China; 4 Department of General Surgery/Pathology, Chinese PLA General Hospital, Beijing, 100853 China; 5 Cancer Center, State Key Lab of Cancer in South China, Sun Yat-sen University, Guangzhou, Guangdong 510275 China

## Abstract

Gastric cancer, like most of other cancers, has an uncontrolled cell cycle regulated by cyclins and cyclin-dependent kinases (CDKs). In this study, we reported that gastric cancer cells showed an accelerated G2/M transition promoted by CREPT/RPRD1B and Aurora kinase B (Aurora B). We found that CREPT/RPRD1B and Aurora B were coordinately expressed during the cell cycle in gastric cancer cells. Deletion of CREPT/RPRD1B disturbed the cell progression and extended the length of cell cycle, leading to a significant accumulation of mitotic cells. Mechanistically, we revealed that CREPT/RPRD1B interacted with Aurora B to regulate the expression of Cyclin B1 in gastric cancer cells. Interestingly, Aurora B phosphorylates S145 in a well-conserved motif of CREPT/RPRD1B. We proposed that phosphorylation of CREPT/RPRD1B by Aurora B is required for promoting the transcription of Cyclin B1, which is critical for the regulation of gastric tumorigenesis. Our study provides a mechanism by which gastric tumor cells maintain their high proliferation rate via coordination of Aurora B and CREPT/RPRD1B on the expression of Cyclin B1. Targeting the interaction of Aurora B and CREPT/RPRD1B might be a strategy for anti-gastric cancer therapy in the future.

## Introduction

Gastric cancer cells show a dysfunctional cell cycle controlled by cyclin-dependent kinases (CDKs) and related cyclins^1^. Mutations and deregulations of genes encoding CDKs and cyclins result in gastric cell cycle dysfunction^2–6^. In both normal and tumor cells, different cyclins and CDKs are activated in different phases during their cell cycles. In particular, Cyclin B1 is highly expressed in G2 phase and reaches its expression peak at the metaphase^7^. Cyclin B1 is responsible for the G2/M transition and the activation of CDK1^8^. At the late G2 phase, Cyclin B1 forms a complex with CDK1 and functions as maturation-promoting factor to promote cells to enter into mitosis^9^. During tumorigenesis, Cyclin B1 is highly expressed in varieties of cancers^10–13^. Reduction of Cyclin B1 results in mitotic defects and tumor suppression^14,15^. However, the detailed mechanism of Cyclin B1 regulation in gastric cancers remains to be elucidated.

Previously, our group reported that CREPT (cell cycle-related and expression-elevated protein in tumor), also named RPRD1B (regulation of nuclear pre-mRNA domain containing protein 1B), promotes cell proliferation and tumor development by altering cell cycle^16^. We have identified that CREPT/RPRD1B regulates the expression of Cyclin D1 in varieties of cancers^16^. Recently, others demonstrated that CREPT/RPRD1B is frequently overexpressed in human endometrial cancers and accelerates cell cycle through up-regulating Cyclin D1, CDK4, and CDK6, main regulators of the G1/S phase transition during cell cycle^17^. Depletion of CREPT/RPRD1B was also found to down-regulate the expression of cell cycle-related genes and then decrease the proliferation and migration of lung cancer cells^18^. All these studies of CREPT/RPRD1B focused on the G1/S phase^16,19,20^; however, it remains unclear whether CREPT/RPRD1B participates in the G2/M phase in gastric cancers.

Aurora kinase B (Aurora B), a serine/threonine kinase, is essential for cell cycle progression especially at the mitotic stage^21^. This kinase functions as an enzymatic core of chromosome passenger complex (CPC), which orchestrates the mitotic process, including chromosome arrangement, histone modification, and cytoplasmic division^22,23^. Recent studies revealed that Aurora B regulates the G2/M phase transition through several key factors at the transcriptional level^19,24,25^. In this study, we observed that Aurora B interacts with CREPT/RPRD1B to up-regulate the transcription of Cyclin B1. We provide evidence that Aurora B phosphorylates CREPT/RPRD1B and the phosphorylated CREPT/RPRD1B plays a critical role for the regulation of Cyclin B1 expression at the G2/M phase.

## Materials and methods

### Plasmids and siRNAs

Myc/HA/Flag-CREPT and its truncations were constructed in this lab. HA-Aurora B and HA-Cyclin B1 were kindly provided by Professor Xing-Zhi Xu, Shen Zhen University, Shenzhen, China. GFP-H2B lentivirus plasmid was provided by Dr. Xue-Min Zhang, Institute of Basic Medical Sciences, National Center of Biomedical Analysis, Beijing, China. The small interfering RNAs (siRNAs) against CREPT were synthesized from GenePharma (Shanghai GenePharma Co. Ltd, China). The CRISPR/Cas9 (clustered regularly interspaced short palindromic repeats/CRISPR-associated 9)-mediated CREPT deletion plasmid was generated based on pSpCas9(BB)−2A-Puro(PX459) vector with guide RNAs (Table S1). CREPT point mutants were constructed using Muta-direct Kit (Saibaisheng, SDM-15, China) in this lab. The primers for construction of the vectors by PCR are presented in Table S1.

### Reagents and antibodies

Thymidine, nocodazole, propidium iodide (PI) and antibodies against β-actin and Flag were purchased from Sigma. Doxycycline was obtained from Clontech. CRYSTAL VIOLET was purchased from Amresco. RO-3306 was purchased from Calbiochem. ProLong Gold antifade reagent was purchased from Life Technology. Antibody against CREPT (3E10) was produced in this lab^26^. Anti-tubulin antibody was purchased from CMCTAG. Anti-Myc (9E10), anti-HA (F-7), anti-Cyclin B1(H-433), anti-Cyclin A (C-19), and anti-Cyclin E (HE12) antibodies were purchased from Santa Cruz Biotechnology. Antibodies against histone H3 and glyceraldehyde 3-phosphate dehydrogenase (GAPDH) were purchased from Cell Signaling Technology. Anti-Aurora B, anti-Cyclin D1, and anti-Cyclin B1 (ab32053) antibodies were purchased from Abcam. Anti-H3S10p antibody was purchased from Millipore. Fluorescent secondary antibodies (goat anti-rabbit IgG and goat anti-mouse IgG) were purchased from Jackson ImmunoReseach.

### Cell culture and transfection

HEK293T, HeLa, and MGC803 cells were cultured in Dulbecco’s modified Eagle’s medium supplemented with 10% fetal bovine serum (FBS). AGS cells was cultured in F-12K medium with 10% FBS. All the cells were kept at 37 °C in a humidified atmosphere with 5% CO_2_. The siRNAs were transfected using Lipofectamine® RNAi-MAX Reagent (Life Technologies, NY, USA). Plasmids were transfected using Vigofect (Vigorous Inc. Beijing, China). To generate the stable cell line, cells were infected by a lentivirus system for overexpression or with the CRISPR/Cas9 vector for CREPT deletion.

### Cell cycle synchronization

Cells were synchronized at the G1/S phase by double thymidine block (DTB) and at the G2/M phase by thymidine-nocodazole. For DTB, cells were treated with 2 mM thymidine for 18 h, released into fresh medium for 9 h, and then treated with 2 mM thymidine again for 16 h. Cells were released and harvested at the indicated time points after being released from DTB. To synchronize cells into the G2/M phase, cells were treated with 2 mM thymidine for 24 h, released for 3 h, and then treated with 340 nM nocodazole for 16 h.

### Luciferase activity, cell viability, and flow cytometric analyses

Luciferase assay was performed in HEK293T and MGC803 cells according to a previous study^20^ with the *CCNB1* promoter reporter constructed by primers *CCNB1*-promoter-F/R (Table S1). Cell viability was measured using Cell Counting Kit-8 (CCK-8, Beyotime, China) according to the manufacturer’s protocol. The primary antibody H3S10p was used for the flow cytometric analysis for the cell mitotic index by a standard protocol^26^.

### Immunoprecipitation, western blot, and immunofluorescence (IF) staining

Indicated antibodies were used for the immunoprecipitation and western blot experiments. IF was performed according to protocol in the lab with H3S10p (1:150) antibody^20^.

### Clinical samples and immunohistochemistry (IHC)

Cancer tissues were collected in the Chinese PLA General Hospital in China. The tissue collection procedure with informed consent was approved by the Clinical Ethic Committee of the Chinese PLA General Hospital. The Tissue Microarrays were purchased from Shanghai Outdo Biotech (Beijing, China). The sections were stained as previously described^27^.

### Time-lapse microscopy imaging and mitotic duration analysis

Cells stably expressing GFP-H2B were seeded in a four-chambered cover glass (Nunc® Lab-Tek® II chambered cover glass, Nunc). Living cell images were collected every 5 min for 16 h by an OLYMPUS IX81-ZDC microscope and analyzed with CellSens Dimension software, Olympus. The duration of mitosis was calculated from nuclear envelope breakdown to cytokinesis finished.

### In vitro kinase assay

Purified glutathione *S*-transferase (GST), GST-CREPT (wild type), or GST-CREPT(S145A) proteins were incubated with Flag-Aurora B protein in a kinase buffer, containing 10 mM HEPES (pH 7.5), 50 mM NaCl, 2 mM MgCl_2_, 1 mM dithiothreitol, 1 mM EGTA, and 0.1 mM ATP. After 30 min at 30 ℃, reactions were stopped by SDS loading buffer. Protein samples were separated by sodium dodecyl sulfate–polyacrylamide gel electrophoresis (SDS-PAGE) and phosphate incorporation was detected by a pan-phosphorylation antibody.

### Statistical analysis

**Fig. 1:**
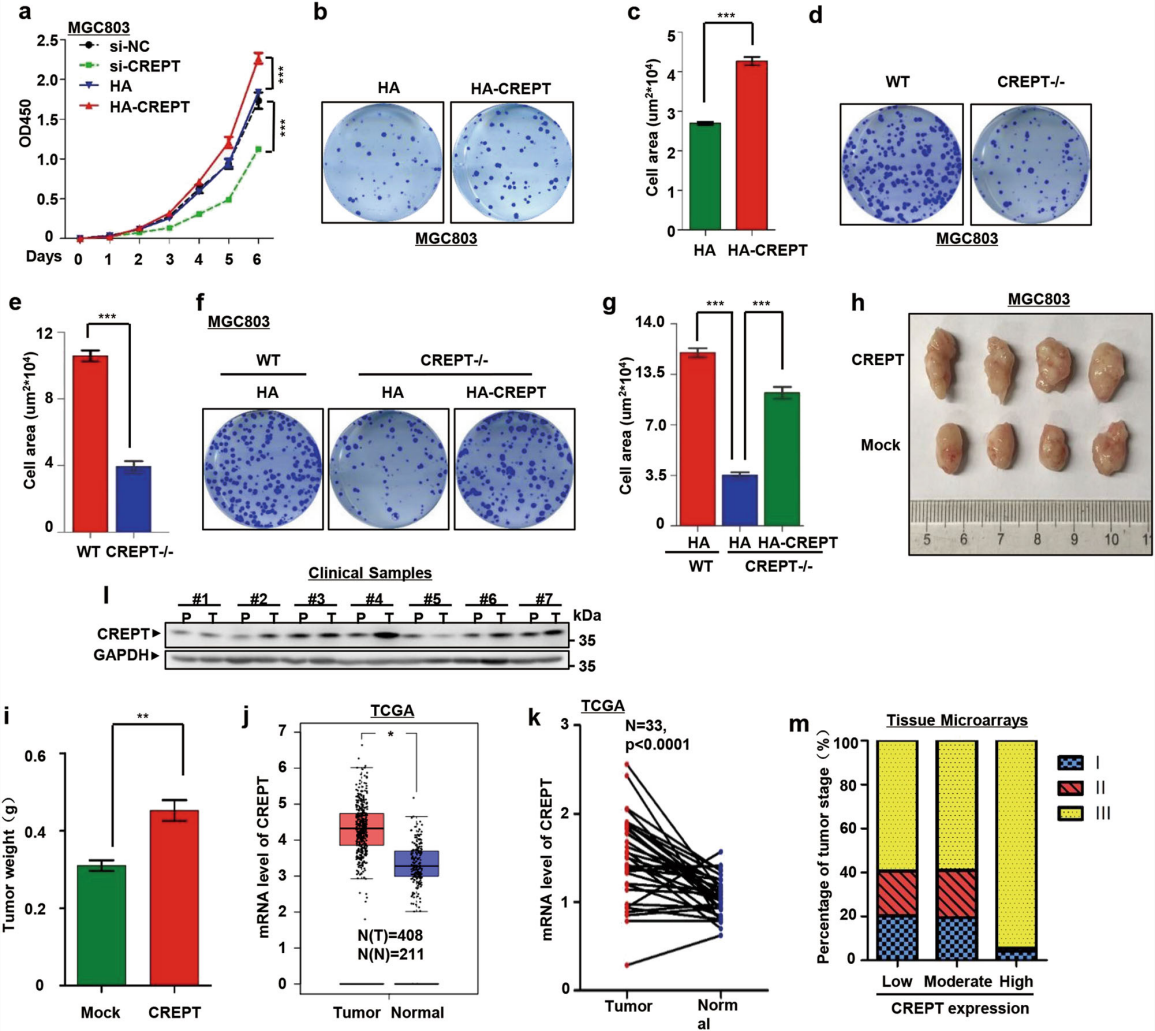
CREPT promotes gastric cancer cell proliferation and is highly expressed in human gastric cancers. **a** CREPT promotes the growth of MGC803 cells. Wild-type (WT), CREPT depletion (si-CREPT), an siRNA vector control (si-NC), CREPT overexpression (HA-CREPT), and vector control (HA) cells were used to measure the cell viability (presented as OD450 values). Results are represented as mean ± SD from three independent repeats. **b**, **c** Overexpression of CREPT promotes colony formation. Colonies formed by MGC803 cells stably overexpressing HA-CREPT were stained (**b**) with crystal violet and counted in three independent experiments (**c**). For each well, 500 cells were seeded and allowed for growth for 10 days. HA presents control cells with empty vector; ***p < 0.001. **d**, **e** Deletion of CREPT inhibits the colony formation. CREPT was deleted by a CRISPR/Cas9 system in MGC803 cells (CREPT–/–). For each well, 1000 cells were seeded and allowed for growth for 10 days. Colonies were stained (**d**) and quantitated from three independent experiments (**e**); ***p < 0.001. **f**, **g** Exogenous expression of CREPT rescues CREPT deletion effect on the colony formation. HA-CREPT was induced into MGC803 cells where endogenous CREPT was deleted by a CRISPR/Cas9 system. For each well, 1000 cells were seeded and colonies were stained (**f**). A quantitative presentation of colonies formation is shown (**g**) from 3 independent experiments; ***p < 0.001. **h**, **i** CREPT promotes the tumor growth. 1 × 10^6^ of mock and CREPT overexpressed MGC803 cells were injected into the flanks of NSG mice (*n* = 4). Mice were killed at day 21 and tumor weight was measured. Data are represented as mean ± SD. **j** CREPT is highly expressed in gastric cancers. Boxplots show the mRNA level of CREPT in tumor and normal tissues. Data were obtained from TCGA and GTEx database; *p < 0.05. **k** Elevated CREPT mRNA is showed in the paired gastric cancer and normal tissues. Levels of CREPT mRNA from paired tumor and normal tissues in the same patients were obtained from TCGA database. **l** The protein level of CREPT is increased in gastric cancers. A western blot was performed for gastric cancer samples from 7 patients. P refers to the paired non-tumor tissue and T refers to the tumor tissue from the same patient. GAPDH was used as a loading control. **m** A statistics of CREPT expression in different gastric cancer stages. Gastric cancers were grouped into three stages and the level of CREPT was categorized as low, moderate, and high from IHC results. Totally, 357 patients were observed

Spearman's correlations of tumor stage and CREPT, CREPT, and Cyclin B1 were estimated (Fig. 1i, 5h, i). The significance analysis of mitosis duration time between wild-type and CREPT deletion cells was performed by Mann–Whitney *U* test (Fig. 4d). Other experiments were subjected to Student’s *t*-test for the significance from at least three independent repeats; ***p < 0.001, **p < 0.01, *p < 0.05. Data were presented as mean ± standard deviation.

## Results

### CREPT promotes gastric cancer cell proliferation and is highly expressed in human gastric cancers

To reveal the role of CREPT in tumorigenesis, we stably overexpressed and depleted CREPT in MGC803 cells, a widely used gastric cancer cell line (Figure S1a). A cell proliferation experiment showed that cells overexpressing CREPT grew faster than the control cells, whereas cells transfected with a mixture of siRNAs against CREPT (Figure S1b) proliferated slowly comparing to the cells transfected with a scramble siRNA (Fig. 1a). Consistently, colony formation assays demonstrated that stably overexpressing CREPT promoted colony formation (Fig. 1b, c), whereas deletion of CREPT by a CRISPR/Cas9 system (Figure S1c) yielded fewer colonies (Fig. 1d, e). To avoid possible defect of overexpression, we determined to express CREPT in different dosages by either transient or doxycycline-induced expression of CREPT. The results showed that low expression of CREPT remained to promote the colony formation (Figure S1l–q). To further validate these observations, we rescued the expression of CREPT by overexpressing HA-CREPT in CREPT deletion cells. The results showed that deletion of CREPT definitely reduced the cell area of colonies, while exogenous expression of HA-CREPT rescued the effect caused by CREPT deletion significantly (Fig. 1f, g). Similar results were observed in AGS cells, another gastric cancer cell line (Figure S1d–k). We also used different dosages of CREPT expression to demonstrate the effect of low level CREPT on the colony formation. The results showed the higher amount of CREPT overexpression and the higher efficiency of rescued colony formation (Figure S1r–t). The tumorigenic capacity of these stable cell lines in NSG mice was then investigated. The tumor size is increased in CREPT overexpressed MGC803 cells compared with the control cells (Fig. 1h, i). These results suggest that CREPT promotes cell proliferation, colony formation, and tumor growth in gastric cancer cells. To address whether CREPT is related to gastric cancers, we analyzed its expression pattern in tumor and normal tissues using data from The Cancer Genome Atlas (TCGA)^28^ and Genotype-Tissue Expression (GTEx) databases (https://commonfund.nih.gov/GTEx/). The result showed that CREPT was highly expressed in tumors compared with normal tissues (Fig. 1j). An analysis based on tumors and the paired normal tissues from the same patient demonstrated a higher level of CREPT expression in tumors (Fig. 1k). To verify the result from these databases, we performed a western blot analysis using clinical samples. The result showed that CREPT was highly expressed in most of gastric tumors compared with the adjacent tissues in 7 patients (Fig. 1l). We further examined CREPT expression by an IHC staining using a tissue microarray with 357 gastric tumor samples. The result showed that most of the patients (94%) in stage III expressed high level of CREPT (Fig. 1m). Pearson's correlation analysis showed a significant correlation of CREPT expression with cancer stages (Figure S1u, Table S2). Taken together, these data suggest that CREPT is highly expressed in human gastric cancers and promotes gastric cancer cell proliferation.

### The expression of CREPT correlates with cell cycle

**Fig. 2:**
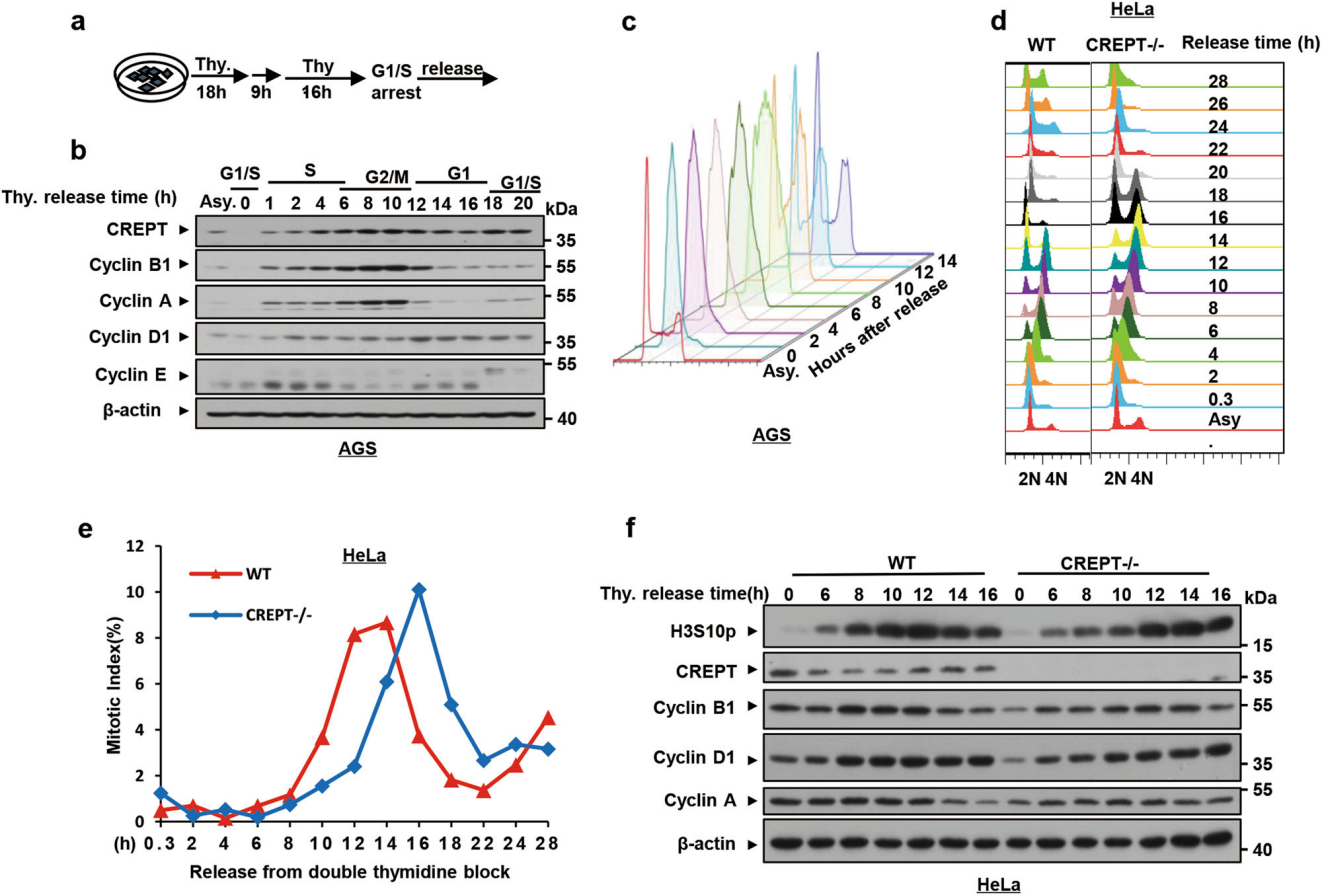
The expression of CREPT correlates with the cell cycle progression. **a** An experimental workflow shows the process of synchronization into G1/S phase. Thymidine (Thy.) was added for 18 h before a 9 h release and was re-added for another 16 h. This double thymidine block (DTB) protocol allows cell synchronized at the G1/S phase. **b** The expression of CREPT alters along with cell cycle stages. The protein level of CREPT was examined by western blots from cells in different cell cycle stages. Asynchronous (Asy.) and synchronized cells were used. Other cell cycle-related proteins were used as markers to indicate the cell cycle stage. β-Actin was used as a loading control. **c** FACS analyses show the cell cycle in different stages after release from DTB. A total number of 2 × 10^4^ cells were used for FACS analyses. **d** CREPT deletion extends the cell cycle. Synchronized wild-type (WT) and CREPT deletion (CREPT–/–) HeLa cells were released and collected at the indicated time points. Cells were stained with PI for FACS analyses. **e** Deletion of CREPT leads to a cell cycle delay of 2 h. A phosphorylation antibody against H3S10p and Alexa Fluor^®^ 488 conjugate secondary antibody were used to analyze the positive cell population by FACS. The mitotic index was presented by the percentage of H3S10p-positive cells. **f** Deletion of CREPT postpones the phosphorylation of H3S10p. A western blot was performed to demonstrate the level of H3S10p for the WT and CREPT–/– cells released from synchronization. Other cell cycle-related proteins were examined to demonstrate the cell cycle correlation

To address how CREPT enhances cell proliferation, we examined the expression of CREPT along cell cycle. For this purpose, AGS cells were synchronized at the G1/S boundary by a DTB protocol and released into different cell cycle stages (Fig. 2a). A western blot analysis showed that CREPT appeared at the late S phase and its expression level increased in the G2/M phase (Fig. 2b, 4–6 h, 8–10 h). The highest level of CREPT corresponded to the highest level of Cyclin B1 and Cyclin A, two cyclin markers for the G2/M phase (Fig. 2b, 8–10 h). Fluorescence-activated cell sorting (FACS) analyses demonstrated a cell cycle change after DTB release (Fig. 2c). These results suggest that the expression of CREPT remains abundant at the G2/M phase.

To address whether CREPT regulates cell cycle, we synchronized the wild-type and CREPT deleted HeLa cells, a typical cell line for studying cell cycle, at the G1/S boundary. FACS analyses indicated that CREPT deletion cells remained in the G2/M phase from 8 to 18 h after being released from DTB, while wild-type cells had already entered into the next G1 phase (Fig. 2d, S2). A quantitative analysis of the mitotic index, presented by phosphorylation level of histone 3 at serine 10 (H3S10p), showed a significant delay of the G2/M phase in CREPT deletion cells (Fig. 2e). Overall, deletion of CREPT appeared to delay the cell cycle for about 2 h (Fig. 2e, see the gap of the curves). A western blot analysis showed that H3S10p peaked at 12 h in wild-type cells, while at 14 h in CREPT deletion cells (Fig. 2f). Taken together, these data indicate that the expression of CREPT correlates with cell cycle and deletion of CREPT extends the G2/M phase.

### Deletion of CREPT leads to significant cell accumulation at G2/M phase and prolongs mitotic progression

**Fig. 3:**
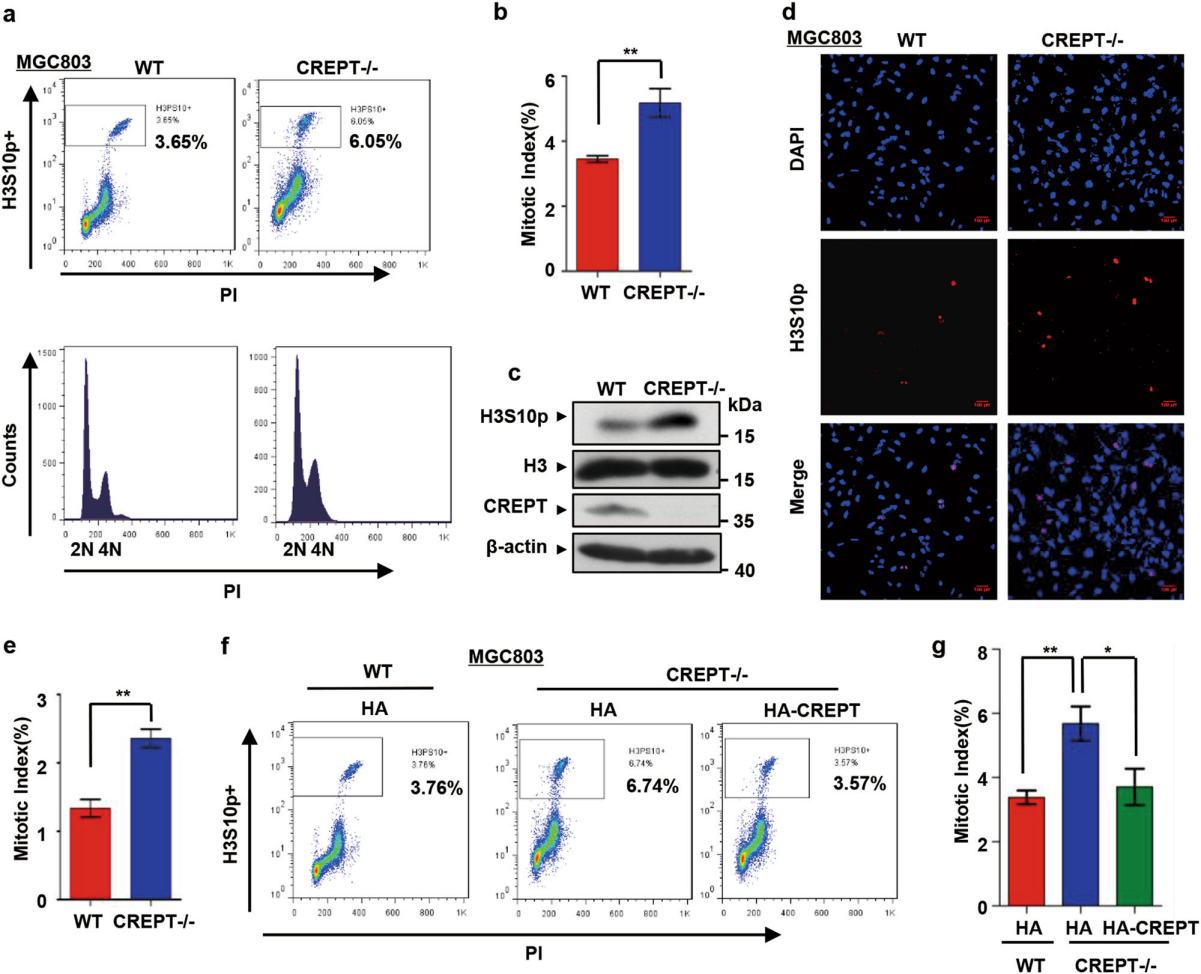
Deletion of CREPT leads to significant mitotic cell accumulation. **a**–**c** The mitotic index increases in CREPT deletion cells. Cells were harvested and stained with PI and H3S10p antibody. FACS analyses showed increased percentage of H3Ser10-positive cells when CREPT was deleted (CREPT–/–) (**a**). A quantitative presentation of the mitotic index from three independent experiments (**b**) **p < 0.01. A western blot showed the level of H3Ser10 phosphorylation. H3 and β-actin were used as loading controls (**c**). **d** Immunofluorescence visualization of mitotic cells. Wild-type (WT) and CREPT deletion (CREPT–/–) MGC803 cells were stained for H3S10p (red) and DAPI (blue). Scale bars, 100 μm. **e** The percentage of H3S10p-positive cells. Approximately 4000 cells were counted per sample in three independent experiments; **p < 0.01. **f** Overexpression of HA-CREPT rescues the CREPT deletion-induced mitotic arrest. HA-CREPT was stably induced into CREPT deletion cells. **g** A quantitative presentation of the mitotic index. Three independent experiments were analyzed by Student's *t*-test; **p < 0.01, *p < 0.05

To investigate the function of CREPT in G2/M progression, we examined the mitotic index in MGC803 cells. A FACS analysis showed that the population of G2/M phase cells increased when CREPT was deleted (Fig. 3a, b, from 3.65% to 6.05% and S3a, b). Consistently, a western blot analysis indicated that the level of H3S10p was increased under CREPT deletion (Fig. 3c). These results were further confirmed by an IF staining analysis (Fig. 3d, e). All these results suggest that CREPT promotes the G2/M progression. To further confirm that the accumulation of G2/M cells is due to the loss of CREPT, we rescued the CREPT-null cells by overexpressing HA-CREPT. The results showed that the mitotic index decreased when HA-CREPT was introduced into CREPT-null cells (Fig. 3f, g and S3c, d). Furthermore, the increased mitotic index in CREPT deleted cells was restored by CREPT overexpression in a dose-dependent manner (Figure S3e, f). Taken together, all the results suggest that deletion of CREPT leads to an accumulation of cells at G2/M phase.

**Fig. 4:**
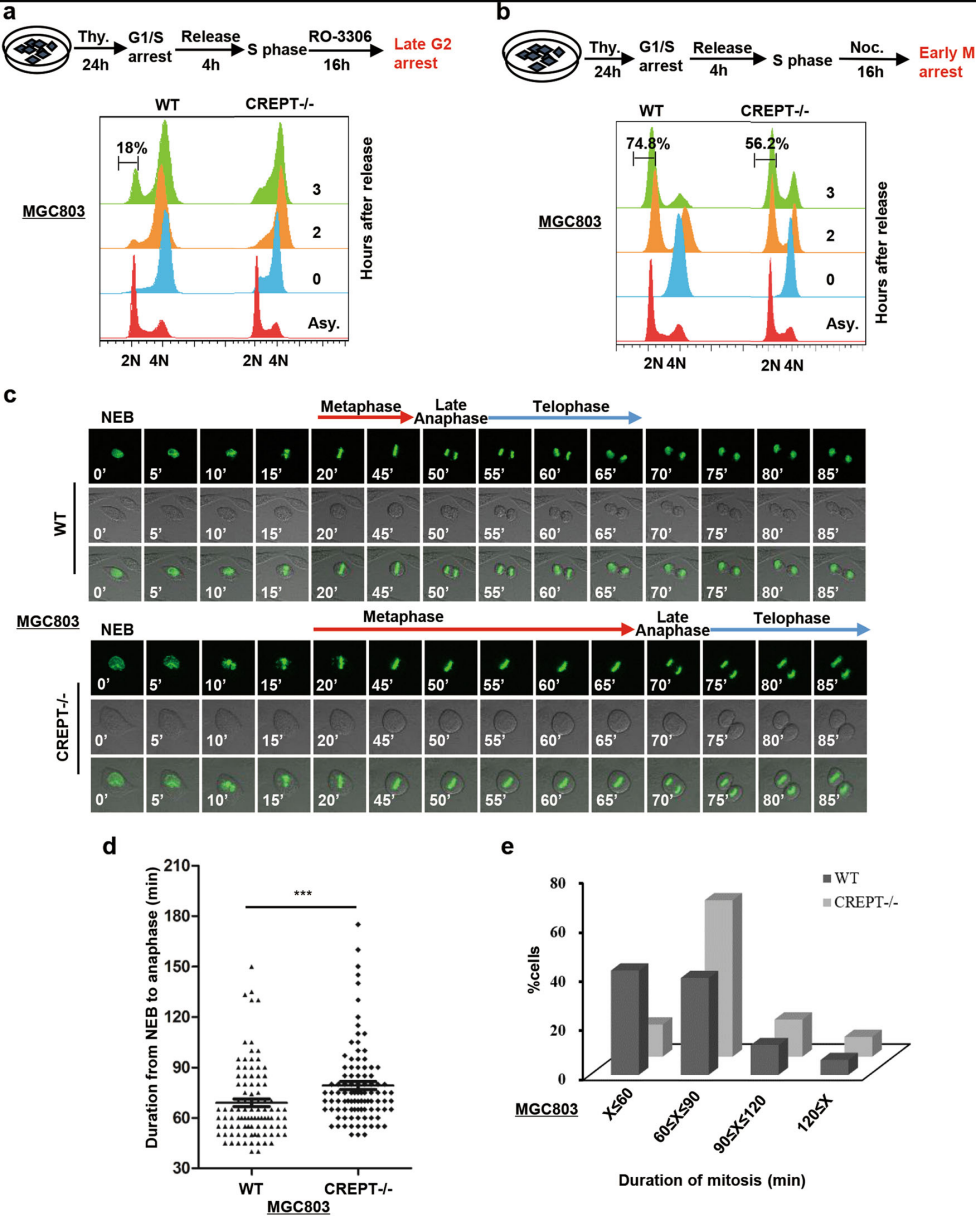
Deletion of CREPT prolongs mitotic progression in gastric cancer cells. **a** An experimental workflow shows the process of synchronization cells into late G2 phase by thymidine (Thy.) and CDK1 inhibitor (RO-3306) (Top). Deletion of CREPT induces cell to arrest at the G2/M phase. Wild-type (WT) and CREPT deletion (CREPT–/–) MGC803 cells were treated by thymidine and RO-3306 and released into normal fresh medium. DNA contents were analyzed by FACS (2 × 10^4^ cells/sample) (bottom). **b** An experimental workflow shows the process of synchronization cells into the early M phase by thymidine (Thy.) and nocodazole (Noc.) (Top). Deletion of CREPT delays the cell entry into the next G1 phase. Thymidine- and nocodazole-treated cells were released and analyzed for DNA content by FACS (2 × 10^4^ cells/sample) (bottom). **c** CREPT deletion cells exhibit prolonged mitosis. Selected frames from a time-lapse microscopy for GFP-H2B infected wild-type (WT) and CREPT deletion (CREPT–/–) MGC803 cells are shown. NEB nuclear envelope breakdown. **d** A dot pot shows the duration from NEB to telophase in MGC803/GFP-H2B cells where CREPT was deleted (CREPT–/–). Wild-type (WT) cells were used as a control; *n* = 99. **e** Distribution of cells during different mitotic stages. About 60% of CREPT deletion cells need about 80 min to finish mitosis, while most of the wild-type cells need less than 60 min. The duration of the mitotic time was categorized into four groups; *n* = 99

To address whether the accumulation of cells at the G2/M phase caused by CREPT deletion is through the prolongation of G2/M process, we synchronized cells at the late G2 phase with thymidine and RO-3306, a CDK1 inhibitor, and allowed the cells to enter into G1 phase (Fig. 4a, top). FACS analyses showed that 18% of wild-type cells entered into the G1 phase at 3 h after release, while most of the CREPT deletion cells remained in the G2/M phase (Fig. 4a, bottom). This result suggests that deletion of CREPT prolongs the duration of G2/M.

To clarify whether deletion of CREPT affects the M phase duration, we synchronized the cells at the early M phase by thymidine and nocodazole (Fig. 4b, top). Results showed that 74.8% of wild-type cells have already entered into the next G1 phase, while only 56.2% of CREPT deletion cells entered into the next G1 phase (Fig. 4b, bottom). Similar results were observed in AGS cells where CREPT was depleted by siRNAs or deleted by CRISPR/Cas9 system (Figure S4). To further analyze the effect of CREPT on the M phase duration, we monitored the mitotic progression by time-lapse imaging in cells stably expressing GFP-tagged histone H2B. The result showed that deletion of CREPT delayed the M phase duration for about 20 min (Fig. 4c). Statistically, we analyzed 99 cells and the average duration of mitosis was found to increase from 70.28 ± 2.57 min in control cells to 80.48 ± 2.67 min in CREPT deletion cells (Fig. 4c). Further analyses indicated that 64.29% of CREPT deletion cells required approximately 80 min to finish cell division while 50% of wild type cells experienced 60 min in the mitosis (Fig. 4e). Together, all these data indicated that down-regulation of CREPT causes the mitotic cell accumulation and prevents transition from the G2/M phase to the next G1 phase.

### CREPT modulates the G2/M duration by regulating Cyclin B1 expression

**Fig. 5:**
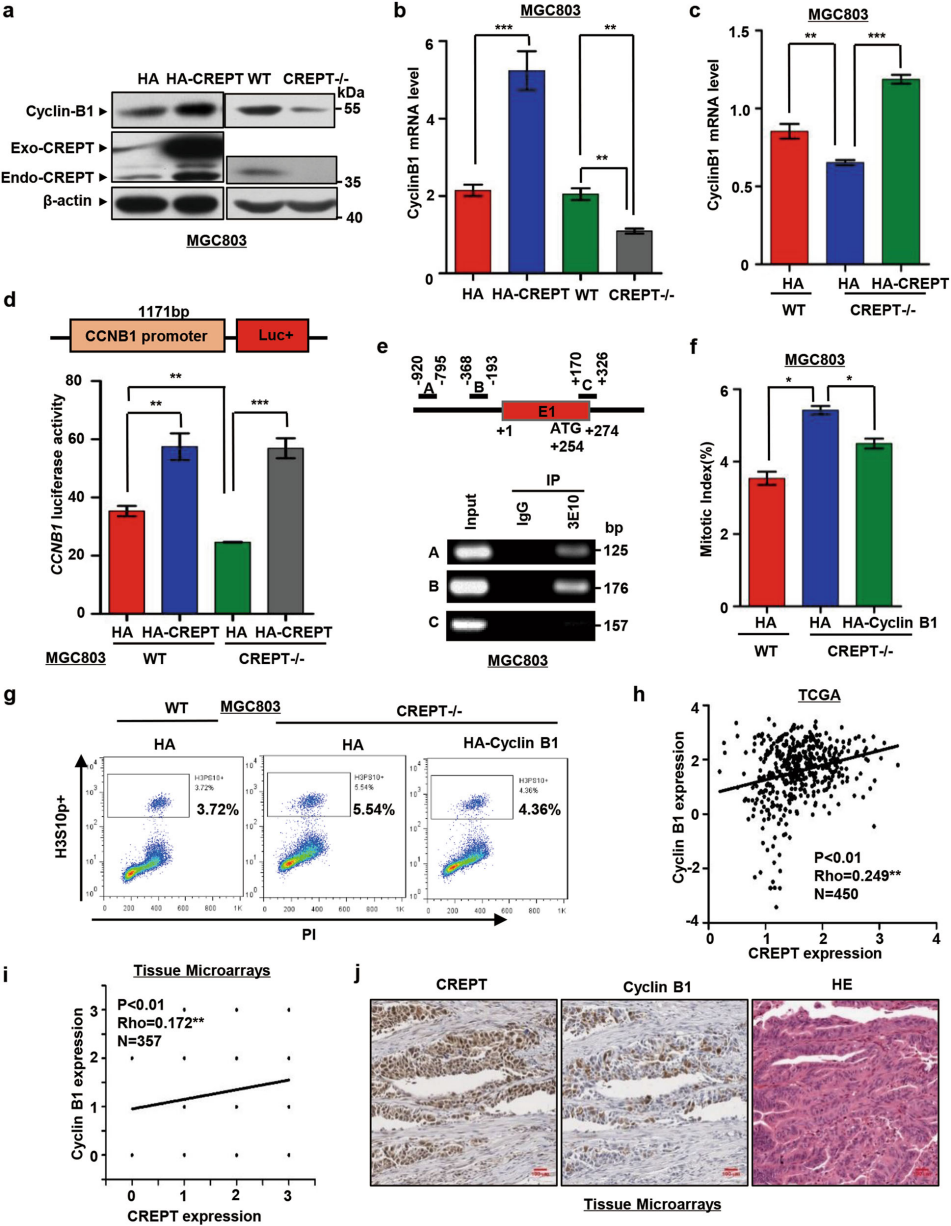
CREPT modulates G2/M arrest by regulating Cyclin B1. **a** Cyclin B1 protein level is increased in CREPT overexpressed MGC803 cells and decreased in CREPT deletion MGC803 cells. Exogenous (Exo-CREPT), endogenous (Endo-CREPT) CREPT and Cyclin B1 levels were examined by western blot. **b** CREPT regulates the expression of Cyclin B1 at mRNA level. Relative mRNA levels of Cyclin B1 were examined using a quantitative real-time PCR from CREPT overexpression (HA-CREPT) or deletion (CREPT–/–) MGC803 cells. The results represent the mean ±  SD from 3 independent experiments; ***p < 0.001, **p < 0.01. **c** Exogenously expressed CREPT restores the decrease of Cyclin B1 in CREPT deletion cells. The level of Cyclin B1 mRNA was examined by a real-time PCR analysis; ***p < 0.001, **p < 0.01. **d** CREPT participates in the transcription of *CCNB1*. An 1171 bp sequence for the *CCNB1* promoter was cloned to drive the expression of luciferase reporter into pGL3-Basic vector (top). Luciferase activities were examined from cells with overexpression of CREPT (HA-CREPT) or deletion of CREPT (CREPT–/–). HA-CREPT was induced to rescue the effect of deletion. The activity was expressed as fold changes, normalized by an internal control (*Renilla*). Results were from three independent repeats and presented as means ±  SD; ***p < 0.01, **p < 0.01. **e** CREPT occupies at the promoter of *CCNB1*. Primers at the *CCNB1* gene are shown. E1 represents exon 1. Numbers indicate the nucleotides counting from the primary translation site as +1 (top). ChIP experiments were performed by immunoprecipitation (IP) with an antibody against CREPT (3E10) and by PCR amplification of the fragments (Bottom). **f** Exogenous expression of Cyclin B1 partially rescues the CREPT-induced mitosis arrest. HA-Cyclin B1 was transiently transfected into CREPT deletion MGC803 cells (CREPT–/–). After 48 h, cells were harvested and stained with PI and H3ser10 antibody and Alexa Fluor® 488 conjugate secondary antibody. The mitotic index was measured by FACS. Three independent experiments were performed and the results were presented as mean ± SD; *p < 0.05. **g** A presentative FACS analysis for the population of mitotic cells. **h**–**j** CREPT correlates with Cyclin B1 expression. Spearman's correlation (Rho) was calculated with 450 gastric cancer samples from the TCGA database (**h**) and a tissue microarray containing 357 gastric cancer samples (**i**). The tissue microarray was stained with anti-CREPT or anti-Cyclin B1 antibody respectively. The expression levels of Cyclin B1 and CREPT were classified as low, moderate, and high according to a DAB staining result. Representative immunohistochemical staining is shown. Scale bars, 100 μm (**j**)

Cyclin B1 is a critical regulatory protein required for the initiation of mitosis and the transition from G2 to M phase^29^. As deletion of CREPT lead to retarded G2/M phase, we speculated that CREPT might function via Cyclin B1. For this purpose, we examined the effect of CREPT on the expression of Cyclin B1. The result showed that overexpression of CREPT increased, while deletion of CREPT reduced the Cyclin B1 protein level (Fig. 5a, S5f). Consistently, the messenger RNA (mRNA) level of Cyclin B1 was up-regulated by stable overexpression of HA-CREPT but down-regulated by deletion of CREPT (Fig. 5b, S5e). Furthermore, overexpression of HA-CREPT in CREPT deletion cells significantly rescued Cyclin B1 mRNA level (Fig. 5c). These results suggest that CREPT regulates the expression of Cyclin B1 at the transcriptional level.

To reveal whether CREPT directly regulates Cyclin B1 transcription, we inserted an 1171 bp genomic sequence upstream of the human *CCNB1* promoter into a luciferase reporter vector (Fig. 5d, top)^30^. A luciferase reporter assay demonstrated that overexpression of CREPT enhanced the luciferase activity while deletion of CREPT impaired this activity (Fig. 5d, bottom and S5a–d). Furthermore, we observed that CREPT occupied on the promoter region of *CCNB1* by a chromatin immunoprecipitation (ChIP) experiment (Fig. 5e). These results indicated that CREPT regulates the Cyclin B1 expression via directly targeting its promoter region during transcription.

To investigate whether Cyclin B1 is responsible for CREPT-associated regulation of G2/M cell accumulation, we overexpressed Cyclin B1 in CREPT deletion cells. Results showed that deletion of CREPT increased the G2/M phase population and exogenous expression of HA-Cyclin B1 partially restored the mitotic index (Fig. 5f, g), suggesting that CREPT regulates the G2/M phase duration through Cyclin B1.

Furthermore, to examine whether CREPT correlates with Cyclin B1 in gastric cancers, we analyzed the expression relationship between CREPT and Cyclin B1 using data from TCGA database^28^. A positive correlation between the expression of CREPT and Cyclin B1 was observed from 450 samples (Fig. 5h). Consistently, the positive correlation of CREPT and Cyclin B1 expression was also confirmed by an IHC experiment with tissue microarrays (Fig. 5i, j, S5g, h, Table S3). All the data indicate that CREPT regulates the transcription of Cyclin B1 in gastric cancers.

### CREPT interacts with Aurora B

**Fig. 6:**
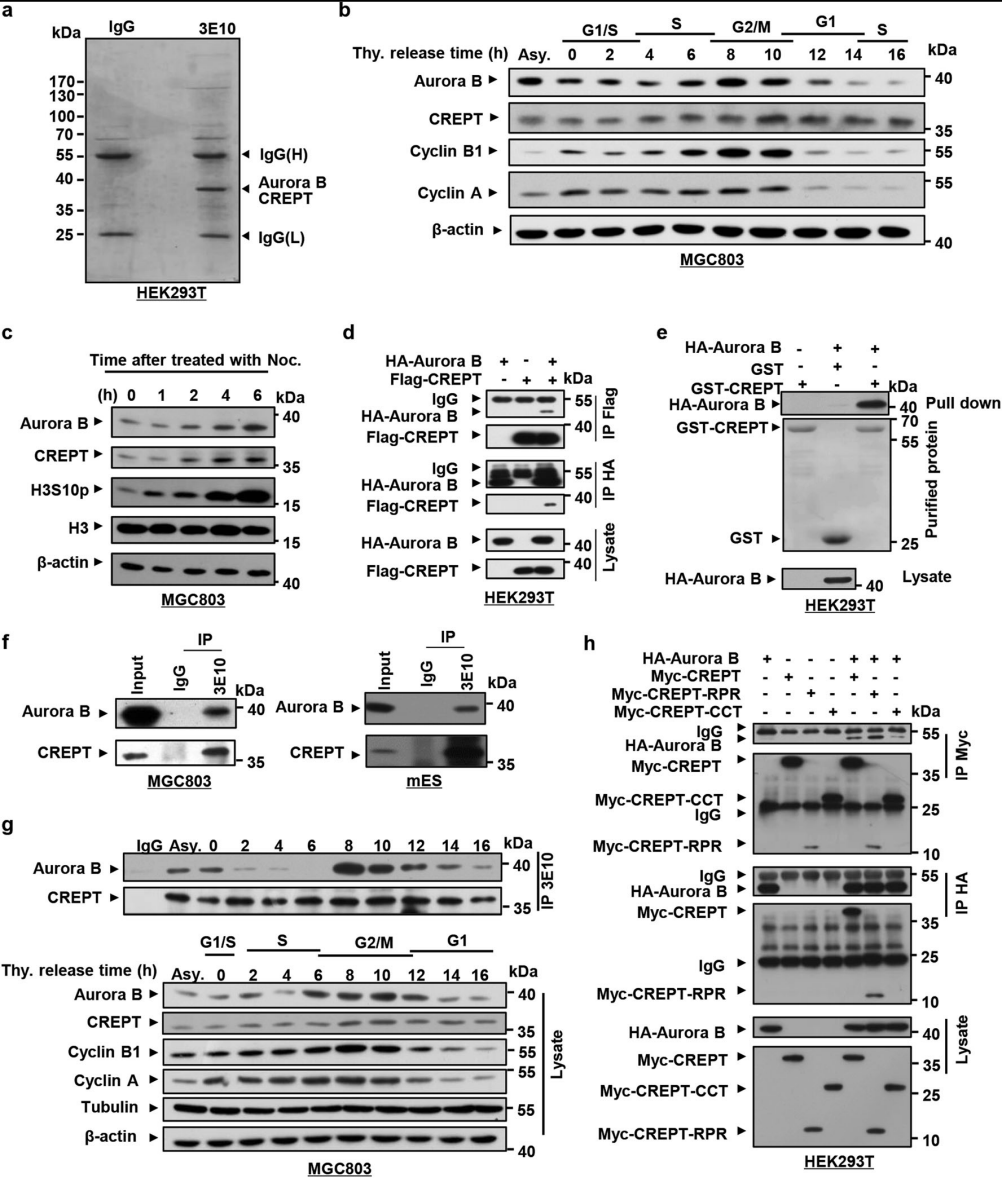
CREPT is associated with Aurora B. **a** A mass spectrometry analysis of proteins associated with CREPT. A mass spectrometry analysis was performed to identify proteins in the complex precipitated by an antibody against CREPT (3E10). IgG was used as a control. Precipitants were analyzed by an SDS-PAGE and stained with Coomassie blue. Bands were cut out and identified by a mass spectrometry analysis. **b** Aurora B and CREPT are coordinately expressed at the G2/M phase. Cells were synchronized by double thymidine blocks and released into complete medium to allow cell entry into different phases. Samples were collected at the indicated time points and mitotic markers were analyzed by western blots. **c** The protein levels of CREPT and Aurora B are both increased in the G2/M stage. Nocodazole was added into medium at indicated time point and the cells were harvested at the same time. **d** CREPT specifically interacts with Aurora B in vitro. Flag-CREPT and HA-Aurora B were transfected into HEK293T cells and harvested for the precipitation after 24–36 h. Immunoprecipitation (IP) was performed using an antibody against Flag or HA. The complex was examined by a western blot using an antibody against HA or Flag. **e** CREPT and Aurora B physically interact in vitro. A GST pull-down assay was performed with purified GST and GST-CREPT proteins. HA-Aurora B was overexpressed in HEK293T cells. **f** Endogenous CREPT interacts with Aurora B in vivo. An IP experiment was performed with an anti-CREPT antibody. Experiments were performed in MGC803 cells and mouse embryonic stem cells (mES). **g** The interaction of Aurora B and CREPT occurs at the G2/M phase. The cells were synchronized by DTB and IP was performed with anti-CREPT antibody at different phases during the cell cycle. **h** CREPT interacts with Aurora B through the RPR domain. HA-Aurora B was co-expressed in HEK293T cells with Myc-tagged full length and two deletions of CREPT. Cell lysates were immunoprecipitated using an anti-Myc or anti-HA antibody

To reveal factors involved in the transcriptional regulation of Cyclin B1 by CREPT during the mitotic progression, we performed a mass spectrometry (MS) analysis from an immunoprecipitation (IP) experiment using an antibody (3E10)^26^ against CREPT (Fig. 6a). Interestingly, we identified Aurora B in the complex. This result echoes the fact that CREPT regulates the G2/M phase where Aurora B functions as a major regulator^21,23,31^. To confirm whether CREPT and Aurora B co-express with Cyclin B1 during the G2/M phase, we examined their expression levels during cell cycle after synchronization. The results showed that Aurora B reached its highest level at the G2/M phase, when CREPT and Cyclin B1 also remained abundant (Fig. 6b). Intriguingly, we observed that both Aurora B and CREPT protein levels increased after cells were treated with nocodazole allowing an arrest at the G2/M phase (Fig. 6c). These results suggest that Aurora B, CREPT, and Cyclin B1 are co-expressed in the G2/M phase.

To question whether CREPT interact with Aurora B, we performed an IP experiment in cells overexpressing HA-Aurora B and Flag-CREPT. The result showed that an antibody against Flag precipitated down HA-Aurora B (Fig. 6d, upper panels) and reciprocally an antibody against HA precipitated down Flag-CREPT (Fig. 6d, lower panels). A GST pull-down assay using GST-CREPT purified from *Escherichia coli* showed that Aurora B directly interacted with CREPT (Fig. 6e). Indeed, we observed that endogenous CREPT and Aurora B associated in MGC803 (Fig. 6f, left) and mouse embryonic stem (mES) cells (Fig. 6f, right). In particular, we observed that the association of CREPT and Aurora B occurred in most of the cell cycle except S phase (Fig. 6g, h). The interaction began to increase sharply from the G2 phase (Fig. 6g, 8 h). These results suggested that Aurora B might play an essential role in the association with CREPT specifically at the G2/M phase. Furthermore, we revealed that the RPR domain of CREPT was critical for the interaction by an IP experiment with Myc-tagged CREPTs and HA-tagged Aurora B (Fig. 6h). Taken together, these results suggest that CREPT interacts with Aurora B in vitro and in vivo.

### Aurora B and CREPT regulate the expression of Cyclin B1

**Fig. 7:**
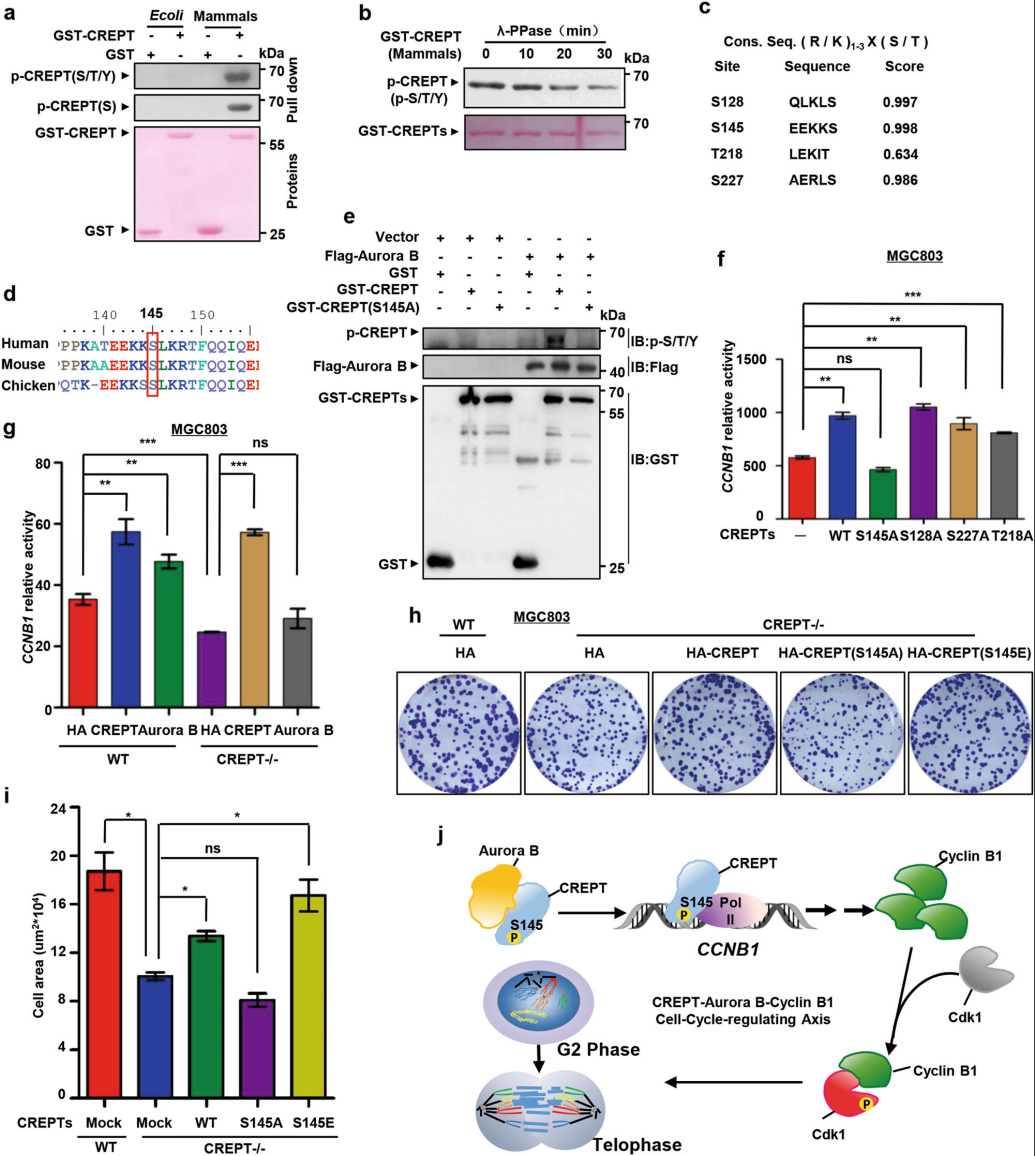
Aurora B and CREPT regulate the expression of Cyclin B1. **a** CREPT is phosphorylated in eukaryotic cells. Purified GST-CREPT in prokaryotic (*E. coli*) and eukaryotic (Mammals) cells were examined using general anti-phosphorylation antibodies by western blots. A pan-phosphorylation antibody, marked as p-CREPT(S/T/Y), and a specific anti-serine phosphorylation antibody, marked as p-CREPT(S), were used. **b** The phosphorylation of CREPT is impaired after adding lambda protein phosphatase (λ-PPase). GST-CREPT purified from eukaryotic cells was incubated with λ-PPase to allow proteins of dephosphorylation. A pan-phosphorylation antibody was used for the western blot. **c** Potential phosphorylation sites in CREPT by Aurora B is predicted. The Consensus phosphorylation sequence by Aurora B is shown. The potential phosphorylation sites were predicted by Netphos 3.1. **d** S145 residue is conserved across different species. Sequences of CREPTs among different species are presented. Red box indicates S145, which is conserved. **e** Aurora B phosphorylates CREPT at S145. Flag-Aurora B was purified by an immunoprecipitation with an anti-Flag antibody from HEK293T cells transfected with an expression vector for Flag-Aurora B. The GST, GST-CREPT, and GST-CREPT(S145A) were purified by GST beads. Phosphorylation of GST or GST-tagged CREPT and its mutant catalyzed by Flag-Aurora B was detected by a general anti-phosphorylation antibody (top). **f** S145 in CREPT is responsible for its activity on the transcriptional regulation of *CCNB1* gene. Wild-type (WT) and different mutants of CREPT were transfected with *CCNB1* promoter-luciferase reporter and pRL-TK plasmids into MGC803 cells. Luciferase activities were measured from three independent experiments; **p < 0.01, ***p < 0.001; ns no significant. **g** Aurora B promotes expression of the Cyclin B1 through CREPT. Luciferase activities were examined for cells with overexpression of CREPT and Aurora B in both wild-type (WT) and CREPT deletion (CREPT–/–) MGC803 cells. Note that overexpression of CREPT rescued but Aurora B failed the activity in CREPT deletion cells. The experiments were performed in three independent repeats; **p < 0.01, ***p < 0.001; ns no significant. **h**–**i** Wild-type CREPT, but not CREPT(S145A), restores the colony formation impaired by endogenous CREPT deletion. Vector and HA-CREPTs were exogenously expressed in MGC803 cells where endogenous CREPT was deleted by a CRISPR/Cas9 system. For each well, 1000 cells were seeded and colonies were stained (**h**). A quantitative presentation of colonies formation is shown (**i**) from 3 independent experiments; *p < 0.05, ns no significant. **j** A model for the regulation of Cyclin B1 expression by CREPT and Aurora B at the G2/M transition. Aurora B interacts with CREPT and phosphorylates CREPT at S145. The phosphorylated CREPT promotes the transcription of Cyclin B1, which promotes the phosphorylation of Cdk1, leading to a quick cell transition from the G2 phase into the M phase to accelerate cell proliferation and metastasis

Since Aurora B is a serine/threonine kinase^21^, we determined to address whether CREPT is phosphorylated by Aurora B. For this purpose, we purified GST-CREPT protein in both prokaryotic and eukaryotic cells to examine the phosphorylation of CREPT using a pan-phosphorylation antibody. The result showed that CREPT is phosphorylated in eukaryotic cells but not in prokaryotic cells (Fig. 7a). An in vitro dephosphorylation experiment by λ-PPase, a classical phosphatase, confirmed the phosphorylation of CREPT (Fig. 7b). These data suggest that phosphorylation of CREPT occurs in eukaryotic cells.

To reveal possible residues responsible for CREPT phosphorylation, we analyzed CREPT protein sequence using NetPhos3.1 (http://www.cbs.dtu.dk/services/NetPhos/) and identified four potential Aurora B phosphorylation sites (S128, S145, T218, and S227) which shared the Aurora B phosphorylation site consensus motif (Fig. 7c). A mass spectrometry analysis identified Ser145 as a phosphorylation site of CREPT in the intact cells (Figure S6a). Interestingly, we found that Ser145 was conserved across different species (Fig. 7d), implying that this residue was important for the function of CREPT. To elucidate whether CREPT was phosphorylated at Ser145 by Aurora B, we performed an in vitro kinase assay using purified Flag-Aurora B together with wild-type or mutated GST-CREPT. We found that Aurora B indeed phosphorylated wild-type CREPT but failed to phosphorylate the S145A mutant (Fig. 7e). Taken together, these results suggest that Aurora B has kinase activity to CREPT at serine 145.

To further investigate whether S145 is essential for CREPT to promote the transcription of Cyclin B1, we performed a luciferase assay by co-transfection of the *CCNB1* promoter reporter with CREPT mutants. The result showed that CREPT(S145A) lost its ability to enhance the transcriptional activity, whereas S128A, T218A, and S227A mutants maintained the activity (Fig. 7f). Moreover, S145E, a constitutively active mutation of S145, appeared to maintain the activity on the transcription of *CCNB1* (Figure S6b). Correspondingly, the interaction between CREPT(S145A) and Aurora B decreased dramatically (Figure S6c). On the other hand, we observed that Aurora B(K106R), a mutant of Aurora B which lost its kinase activity^32^, failed to interact with CREPT (Figure S6d), suggesting that the kinase activity is required for the interaction of Aurora B and CREPT. All these results suggest that phosphorylation of S145 is required for CREPT in promoting the transcription of Cyclin B1.

To examine whether Aurora B depends on CREPT in promoting the transcription of Cyclin B1, we co-transfected HA-CREPT or HA-Aurora B with the *CCNB1* promoter reporter in CREPT deletion cells. The results showed that HA-CREPT restored the reporter activity while Aurora B failed in CREPT deletion cells (Fig. 7g). On the other hand, inhibitors against Aurora B inhibited the *CCNB1* promoter activity (Figure S7a) and the protein level of Cyclin B1 (Figure S7b). Correspondingly, the inhibitor against Aurora B inhibited the colony formation of the MGC803 cells (Figure S7c). These results suggest that Aurora B regulates the transcription of Cyclin B1 depending on CREPT.

To further evaluate the role of CREPT phosphorylation on the colony formation and cell mitosis, we stably overexpressed CREPT and its mutants in CREPT deleted MGC803 cells (Figure S7d). The results showed that overexpression of CREPT(S145A) failed to promote the colony formation (Figure S7e, f) and tumor metastasis (Figure S7g), whereas CREPT(S145E) maintained these abilities. Furthermore, we observed that wild-type CREPT rescued the decreased colony formation (Fig. 7h, i) and the mitotic index (Figure S7h, i) caused by CREPT deletion, but CREPT(S145A) failed. Taken together, all these results demonstrate that S145 is essential for CREPT to regulate cell cycle and to promote cell proliferation and metastasis (Fig. 7j).

## Discussion

We have reported that CREPT is a cell cycle-related oncogene highly expressed in tumors and involved in tumor development^16–18^. However, how CREPT regulates the cell cycle during tumorigenesis is still elusive, although we demonstrated that CREPT regulated Cyclin D1 expression^16^. In this study, we provided evidence that CREPT regulated the G2/M transition by up-regulating Cyclin B1 expression in gastric cancer cells. Interestingly, we revealed that Aurora B interacted with CREPT to mediate its phosphorylation. As Cyclin B1 is highly expressed in a variety of cancers, leading to uncontrolled cell proliferation^33^, our results explain why the expression of Cyclin B1 is elevated during tumorigenesis. We believe that Aurora B-driven CREPT phosphorylation is critical for the Cyclin B1 expression in gastric cancers (Fig. 7j).

Previously, we reported that CREPT regulates the expression of Cyclin D1, which is a major regulator for the G1 phase^16^. Others also confirmed our results for the role of CREPT in G1 phase^17,18^. In this study, we observed that CREPT functions in G2 phase, where it regulates the expression of Cyclin B1. Our data consistently provided evidence that CREPT is highly expressed in gastric tumors where Cyclin B1 is overexpressed (Fig. 5h–j). This study extended the role of CREPT in not only G1 but also G2 phase. In our experiments, we overexpressed CREPT at different dosages so that it is comparable for the expression to the clinical samples. In particular, we observed that low level of exogenous expression of CREPT remained to enhance the cell colony formation (Figure S1l–t), rescue the mitotic arrest caused by endogenous CREPT deletion (Figure S3e, f), and promote Cyclin B1 expression (Figure S5c–f). These results confirmed the role of CREPT in the tumorigenesis at the pathological level. Overall, we have demonstrated that CREPT regulates Cyclin D1 and Cyclin B1 to promote tumorigenesis. However, we could not exclude other possibilities that CREPT may regulate different gene expression during the tumorigenesis.

The Cyclin B1/CDK1 complex forms at the border of G2/M phase to allow cells entering into the M phase and maintaining the M phase progress. Our data demonstrated that deletion of CREPT delayed the duration of mitosis for about 20 min (Fig. 4) and the whole G2/M phase for 2 h (Fig. 2d). Moreover, we observed that CREPT mainly associated with Aurora B at the G2/M phase. Cyclin B1 has been proved to function in multiple steps during mitosis; however, we did not observe any change of other phases after metaphase when CREPT was deleted (Fig. 4c). Our results suggest that the role of CREPT on mitosis via promoting Cyclin B1 expression mainly occurs at G2 and early M phase. We reasoned that the 20 min delay in metaphase might be due to other factors that CREPT may associate in the spindle assembly check-point, where Cyclin B1 is needed to be degraded. The detailed mechanism for this Cyclin B1-independent role of CREPT during metaphase process requires further studies.

Aurora B functions in mitotic events to directly regulate the assembly of chromosome^34^, the central spindle assembly and cleavage furrow ingression^23^. However, in this study, we revealed a role of Aurora B in the regulation of Cyclin B1 transcription at the G2 and early M phases. Since transcription is suppressed during mitosis^35,36^, we speculate that the involvement of Aurora B in the regulation of transcription is different from its role in the regulation of mitotic events. This difference might be caused by the different substrates Aurora B recognized during each stage of the cell cycle. The substrates of Aurora B have been extended to histone proteins, transcriptional factors, cytoskeletal proteins, and enzymes besides its conventional spindle check-point partners^21^. Aurora B also phosphorylates p53 and YY1^25,32^. Interestingly, Aurora B is found to phosphorylate Oct4 extensively at serine 229, leading to its dissociation from chromatin^37^. These studies support our findings that Aurora B interacts with CREPT to regulate the transcription of Cyclin B1 at the G2 phase, although further confirmation is needed.

By mutagenesis analyses, we confirmed that the motif EEKKS was the right target for Aurora B (Fig. 7). Mutation at S145 abolished the ability of CREPT in promoting the transcriptional expression of Cyclin B1. This motif is typically the one that Aurora B recognizes, although we cannot exclude whether Aurora A associates with CREPT. As inhibitors against Aurora kinases have been developed for the therapy of cancers, we speculate that blocking the residue of CREPT that Aurora B recognized could be a strategy for the development of anti-tumor drugs.

## Electronic supplementary material

Supplementary material
