# Supplementary material for: CREPT/RPRD1B associates with Aurora B to regulate Cyclin B1 expression for accelerating the G2/M transition in gastric cancer
Source: Cell Death Dis. 2018 Dec 5;9(12):1172. doi: 10.1038/s41419-018-1211-8 (PMC6281615; doi:10.1038/s41419-018-1211-8)
Supplement: Supplementary file 1 — Supplementary material [file 41419_2018_1211_MOESM1_ESM.pdf]

Supplemental Information

CREPT/RPRD1B Associates with Aurora B to Regulate Cyclin B1 Expression for Accelerating the G2/M Transition in Gastric Cancers

Lidan Ding[1*], Liu Yang[1*], Yuqi He[2*], Bingtao Zhu[1], Fangli Ren[1], Xuanzi Fan[1],Yinyin Wang[1], Mengdi Li[1], Jun Li[3], Yanshen Kuang[4], Sihan Liu[1], Wanli Zhai[1], Danhui Ma[1], Yanfang Ju[4], Quentin Liu[5], Baoqing Jia[4], Jianqiu Sheng[2#], Zhijie Chang[1#]

Supplemental Information Inventory

Figure S1. CREPT Promotes cell proliferation in cancer cells.

Figure S2. Deletion of CREPT alters the cell cycle.

Figure S3. Deletion of CREPT leads to significant mitotic cell accumulation in cancer cells.

Figure S4. Depletion of CREPT prolongs the mitotic progression in cancer cells.

Figure S5. CREPT regulates Cyclin B1 expression and shows a positive correlation with Cyclin B1 in human gastric cancers.

Figure S6. S145 is identified phosphorylated and is crucial for the transcription enhancement on *CCNB1* by an interaction with Aurora B.

Figure S7. Inhibiting Aurora B impairs the *CCNB1* transcriptional activity and S145 phosphorylation is essential for CREPT in colony formation, tumor metastasis and mitosis.

Table S1. Primers used in this paper.

Table S2. A correlation of CREPT expression and gastric tumor stages.

Table S3. A correlation of CREPT expression and Cyclin B1 expression in gastric cancer tissues.

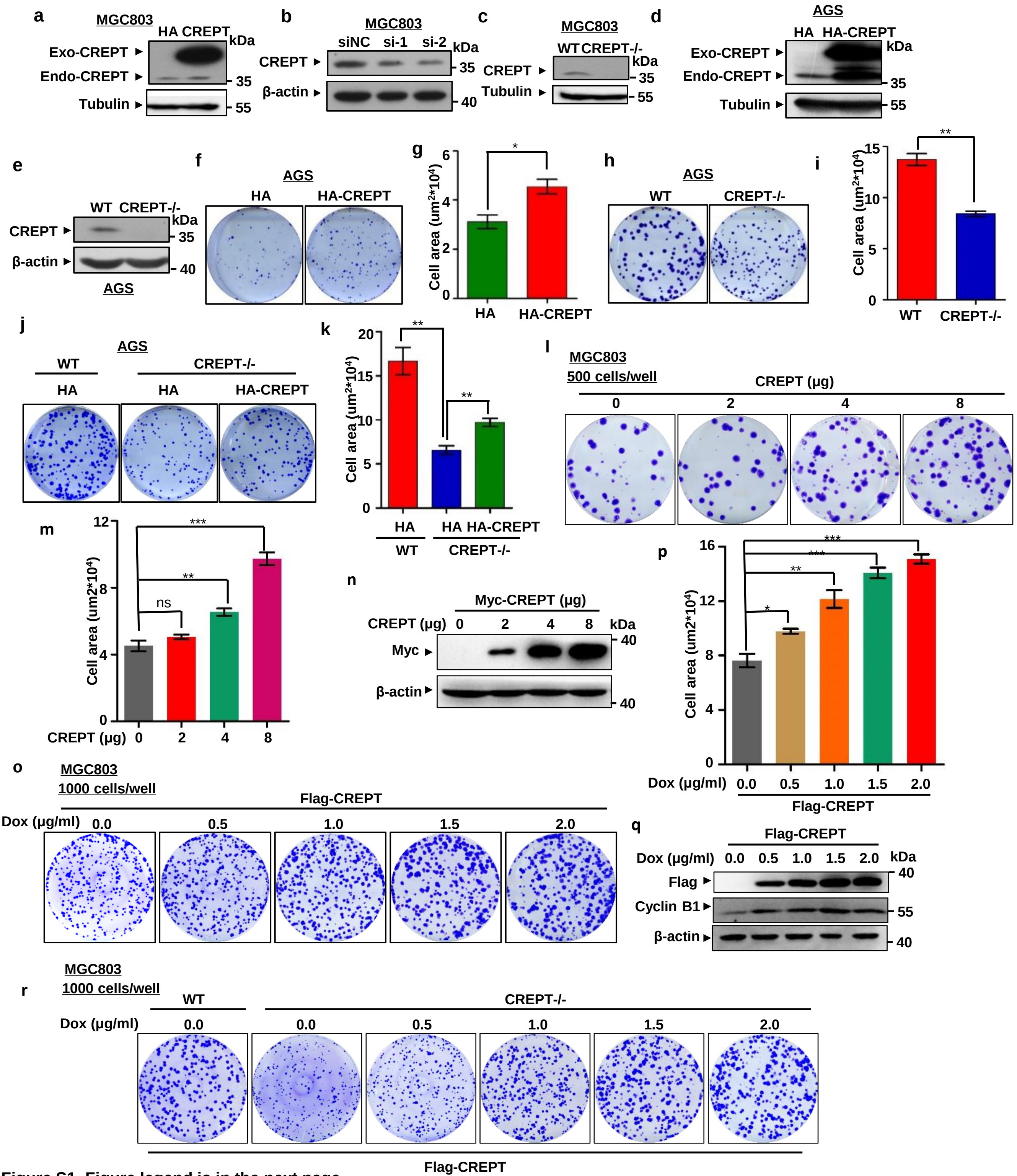

Figure S1. Figure legend is in the next page.

continued

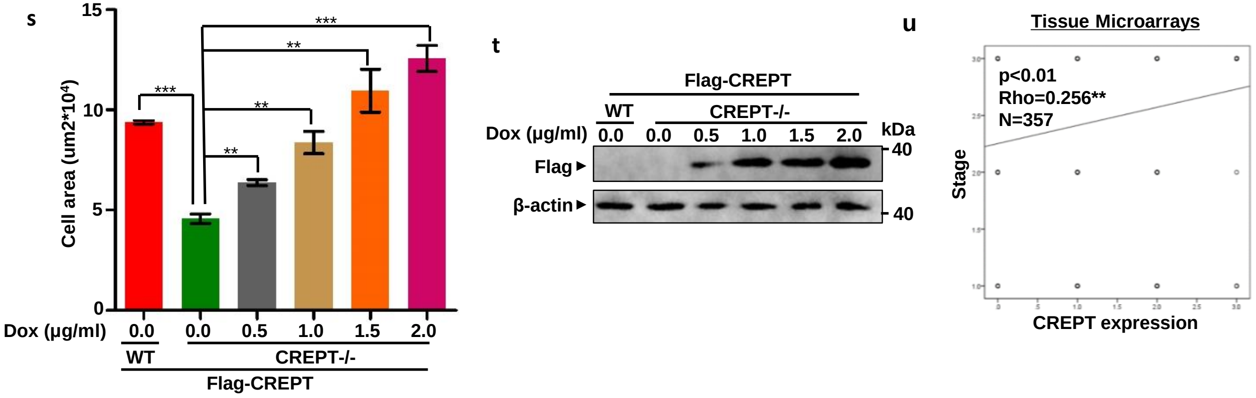

Figure S1. CREPT Promotes cell proliferation in cancer cells.

(**a**) An MGC803 cell-based stable cell line for overexpression of CREPT. A Western blot shows the exogenous (Exo-CREPT) and endogenous (Endo-CREPT) CREPT expression in the stable cell lines which were established by lentivirus infection. HA indicated an empty vector with HA tag. Tubulin was used as a loading control. (**b**) Stable depletion of CREPT. CREPT was depleted by siRNAs in MGC803 cells. Cells were transfected with two siRNAs against CREPT (si-1 and si-2). Non-specific RNAs (siNC) was used as a negative control. Actin was used as a loading control. A Western blot shows the expression of CREPT at protein level. (**c**) A stable cell line for CREPT deletion. MGC803 cells were used to genetically delete CREPT by a CRISPR/Cas9 system. A Western blot using an antibody against CREPT showed the complete deletion of endogenous expression of CREPT. (**d**) A CREPT overexpression cell line based on AGS cells. The exogenous (Exo-CREPT) and endogenous (Endo-CREPT) CREPT expression was examined by a Western blot. (**e**) Deletion of CREPT in AGS cells. Endogenous CREPT was examined by a Western blot. (**f-g**) Overexpression of CREPT promotes colony formation in AGS cells. Colony formation assays were performed in AGS cells where CREPT was stably overexpressed. Cells with overexpression of an empty vector (HA) was used as a control. A quantitative presentation of the colonies formed from three independent experiments based on AGS cells (**g**). (**h-i**) Deletion of CREPT inhibits colony formation in AGS cells. Wild type (WT) and CREPT deletion (CREPT-/-) cells based on AGS cell line were examined. A quantitative presentation of colonies formed from the AGS based cells from three independent experiments is showed (**i**). (**j-k**) Exogenous expression of CREPT rescues the effect of CREPT deletion on the impaired colony formation. HA-CREPT was induced into AGS cells where endogenous CREPT was deleted. A quantitative presentation of colonies formed from the AGS cells is showed. All the quantitative experiments were performed from three independent repeats. T test was used for the significance. *, $p<0.05$, **, $p< 0.01$. (**l-n**) Overexpression of CREPT promotes the colony formation in MGC803 cells. Cells were firstly transfected with different dosages of Myc tagged CREPT in 60 mm dishes and 500 cells of each group were seeded into 6-well plates. After 11 days, the colonies were stained with crystal violet (**l**) and counted in three independent experiments (**m**). *** $p<0.01$, **$p<0.01$. The overexpressed CREPT was examined by a Western blot assay and β-actin was used as loading control (**n**). (**o-q**) Overexpression of CREPT enhances the colony formation ability of MGC803 cells. CREPT coding sequence was inserted into pCW-Flag, a doxycycline induced overexpression vector and a stable cell line containing pCW-Flag-CREPT was established by lenti-virus infection. Different dosages of doxycycline were used to induce CREPT expression at different levels. 1000 cells were seeded into 6-well plates and colonies were stained after 10 days (**o**). Results were represented as mean +/- SD from 3 independent experiments. ***$p<0.001$, **$p<0.01$, *$p<0.05$ (**p**). The protein levels of Flag-CREPT and Cyclin B1 were analyzed by a Western blot and β-actin was used as loading control (**q**). (**r-t**) Overexpression of CREPT rescues the decrease of colony formation ability caused by CREPT deletion in MGC803 cells. The wild type and CREPT deletion cells stably overexpressed exogenous CREPT after doxycycline was added. 1000 cells were seeded into 6-well plates and medium containing different concentrations of doxycycline were changed every two days. Colonies were stained with crystal violet 10 days later (**r**) and results were represented as mean +/- SD from 3 independent experiments. ***$p<0.001$, **$p<0.01$ (**s**). The Western blot results were used to indicate the expression levels of CREPT (**t**). (**u**) CREPT expression correlates with the progress of tumors. Spearman's Rho was calculated from 357 gastric tissues examined by IHC.

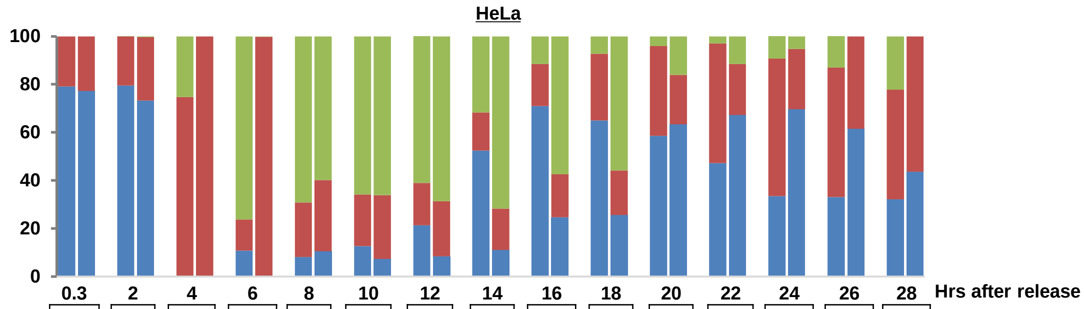

Hrs after release	0.3 WT	0.3 KO	2 WT	2 KO	4 WT	4 KO	6 WT	6 KO	8 WT	8 KO	10 WT	10 KO	12 WT	12 KO
G1	79	77	80	73	0	0	11	0	8	11	13	7	21	8
S	21	23	20	26	75	100	13	100	23	30	22	27	18	23
G2/M	0	0	0	0	25	0	76	0	69	60	66	66	61	69

Hrs after release	14 WT	14 KO	16 WT	16 KO	18 WT	18 KO	20 WT	20 KO	22 WT	22 KO	24 WT	24 KO	26 WT	26 KO	28 WT	28 KO
G1	53	11	71	25	65	26	59	63	47	67	34	70	33	62	32	44
S	16	17	18	18	28	19	37	21	50	21	57	25	54	39	46	56
G2/M	32	72	12	57	7	56	4	16	3	12	9	5	13	0	22	0

Figure S2. Deletion of CREPT alters the cell cycle.

Histograms of proportions of thymidine-treated wild type and CREPT deletion cells at various stages in the cell cycle. Wild type (WT) and CREPT deletion (KO) cells were synchronized at the G1/S boundary by double thymidine block. Cells were harvested at the indicated time point after thymidine release at different times. Cells were stained with PI and DNA contents were analyzed by FACS. The table represents the percentage of the cell stages. Blue, G1 phase, Red, S phase. Green, G2/M phase.

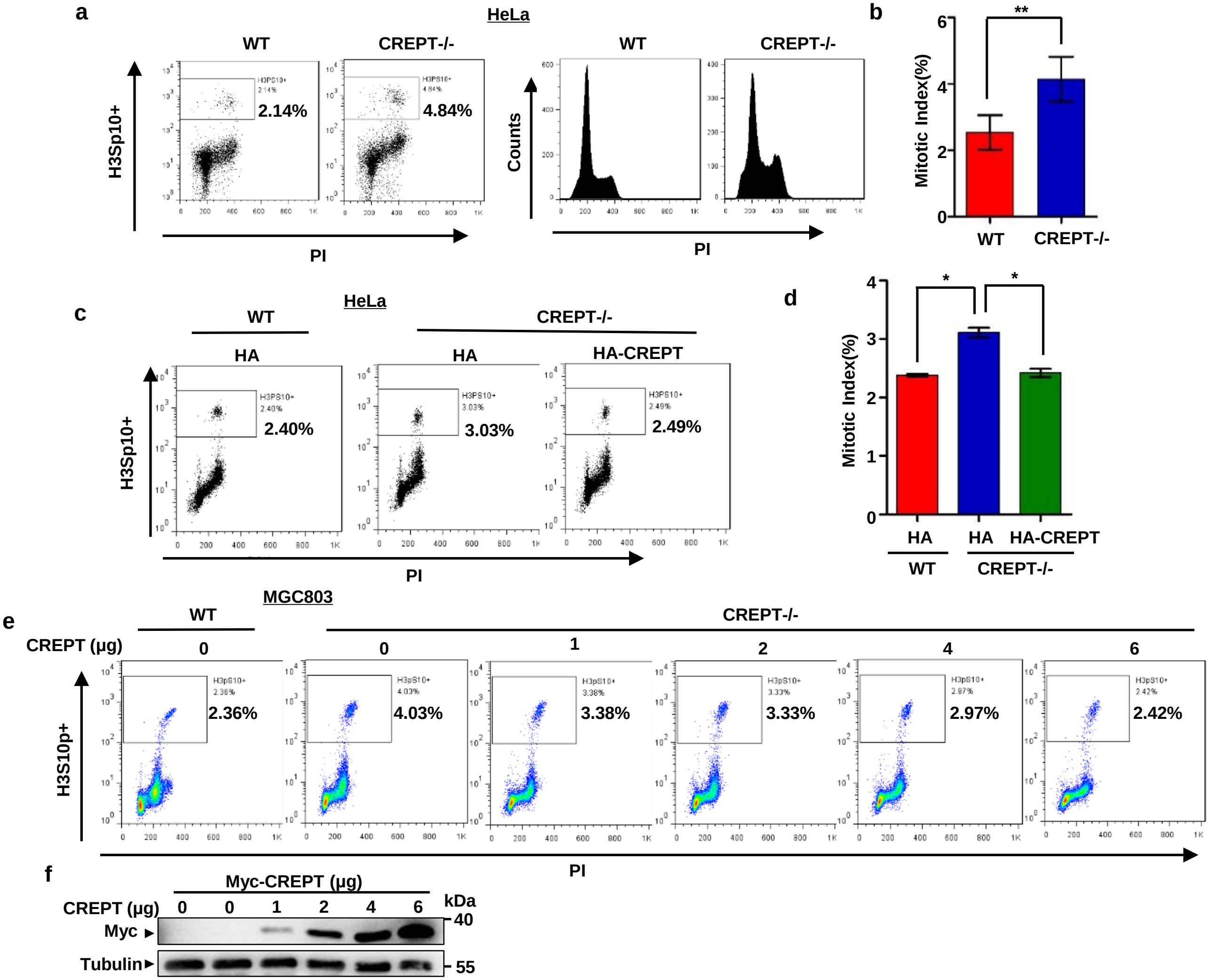

Figure S3. Deletion of CREPT leads to significant mitotic cell accumulation in cancer cells.

(**a**) The mitotic index is increased in CREPT deleted Hela cells. Wild type (WT) and CREPT deletion (CREPT-/-) HeLa cells were harvested and stained with PI and as H3Ser10 phosphorylation antibody. The H3Sp10 positive cells were analyzed by FACS, shown as dots in the inner box (left). Cell cycle was analyzed (right). (**b**) The average mitotic index in the wild type and CREPT deletion cells. Three independent experiments were performed in Hela cells where CREPT was deleted by a CRISPR-Cas9 system. The mitotic index was analyzed by FASC for H3Ser10 phosphorylation positive cells (H3Sp10+ percentage). A student-t test was used to statistically analyze the difference. (**c-d**) Overexpression of CREPT rescues the CREPT deletion induced mitotic arrest. HA-CREPT was stably overexpressed in CREPT deletion (CREPT-/-) Hela cells. FASC was performed to analyze H3Sp10+ cell population (inner box) (**c**). The average mitotic index was calculated from three independent experiments (**d**). In all the quantitative analyses, student t-test was performed. **,p<0.01. *, p<0.05. (**e-f**) The mitotic index increases in CREPT deleted MGC803 cells, and is restored by CREPT overexpression in a dose dependent manner. Different dosages of Myc-CREPT were transfected into wild type and CREPT deleted MGC803 cells and the mitotic indexes (H3Ser10 positive cells) were analyzed by FACS (**e**). A Western blot was used to examine the levels of overexpressed Myc-CREPT and tubulin was used as loading control (**f**).

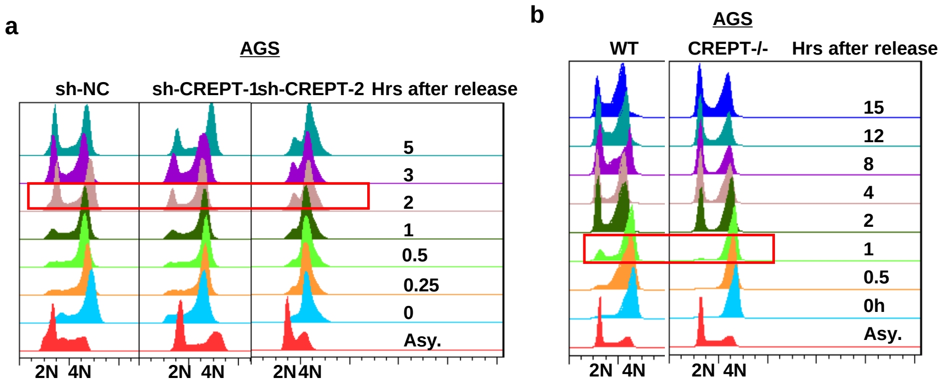

c

Hypothesis Test Summary

	Null Hypothesis	Test	Sig.	Decision
1	The distribution of data is the same across categories of group.	Independent-Samples Mann-Whitney U Test	.000	Reject the null hypothesis.

Asymptotic significances are displayed. The significance level is .05.

Figure S4. Depletion of CREPT prolongs the mitotic progression in cancer cells.

(**a**) Depletion of CREPT delays the cell entry into the next G1 phase in AGS cells. Cells were synchronized by thymidine and nocodazole treatments. Cells were released to different stages and analyzed for the DNA content (2N, 4N) by FACS. Asymmetric (Asy) cells were used as a control. CREPT was depleted by two shRNAs against CREPT (sh-CREPT-1 and sh-CREPT-2). A non-specific shRNA (sh-NC) was used a control. Significant difference of the cell cycles between the control cell (sh-NC) and CREPT depletion cells (sh-CREPT-1 and sh-CREPT-2) is marked by a red box at 2 h after the cells were released to enter the cell cycle. (**b**) CREPT deleted AGS cells exhibit prolonged G2/M stage. CREPT deletion cell line was generated by a CRISPR/Cas9 system. Cells were synchronized by thymidine and nocodazole at the G2/M stage. Samples were harvested at indicated time points for FACS analysis. The histogram of 2N represents the G1 stage and 4N means the G2/M stage. The significant difference of the cell cycles between the control cells and CREPT deletion cells is marked in a red box at 1 h after the cells were released to enter the cell cycle.(**c**) The Mann-Whitney analysis of the data in Figure 4d. (**c**) The Mann-Whitney test for the Figure 4d.

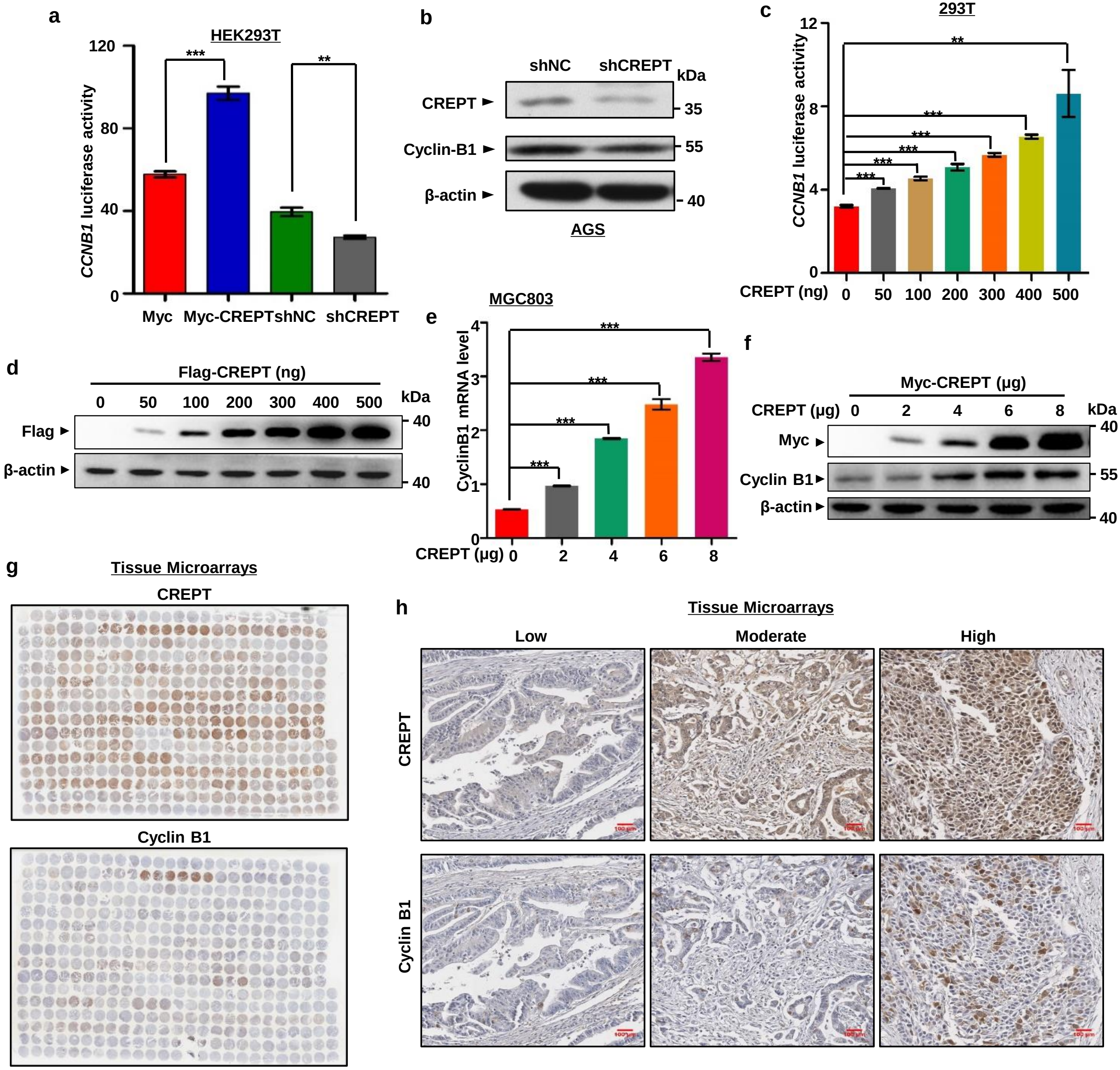

Figure S5. Figure legend is in the next page.

Figure S5. CREPT regulates Cyclin B1 expression and shows a positive correlation with Cyclin B1 in human gastric cancers.

(**a**) CREPT regulates Cyclin B1 expression. Overexpression of CREPT enhanced the transcriptional activity of the *CCNB1* promoter (second column), whereas, depletion of CREPT resulted in a decreased transcriptional activity of the *CCNB1* promoter (last column) in HEK293T cells. *CCNB1* promoter driven luciferase reporter and pRL-TK plasmids were co-transfected into HEK293T cells with Myc-CREPT and Myc-vector. The activity was expressed as fold changes, normalized by an internal control (renilla). Results were from three independent repeats and are presented as means +/- SD. ** $p<0.05$,*** $p<0.001$. (**b**) Cyclin B1 protein level is decreased in CREPT depletion cells. CREPT stably depleted cell line was generated by a lentivirus system infected by a mixture of shRNAs virus against CREPT. A vector control (shNC) and CREPT depletion (shCREPT) were used to generate stable cell line and protein levels were detected by a Western blot. (**c-d**) CREPT promotes the transcription activity of *CCNB1* in a dose dependent manner. Different dosages of Flag tagged CREPT were co-transfected with a *CCNB*1 reporter and an internal control vector (PRL-TK) into HEK293T cells seeded in 24-well plates. Luciferase activities were examined and normalized by an internal control (Renilla). Results were from three independent repeats and presented as means +/- SD. *** $p<0.01$, **$p<0.01$ (**c**). The expression levels of CREPT were determined by Western blot assay and β-actin was used as loading control (**d**). (**e-f**) CREPT regulates the mRNA and protein levels of Cyclin B1 in a dose dependent manner. Different amounts of Myc-CREPT were transfected into MGC803 cells seeded in 60 mm dishes. Relative mRNA levels of Cyclin B1 were examined using a quantitative real-time PCR (**e**) and the protein levels of Cyclin B1 were tested by Western blot (**f**). The mRNA results were represented as mean +/- SD from 3 independent experiments. ***$p<0.001$. (**g**) Immunohistochemical staining of CREPT and Cyclin B1 expression in the gastric cancer tissue microarray. Two sequential gastric cancer tissue assays were used. Totally, 457 cases were involved. (**h**) Representative images of immunohistochemical staining of CREPT and Cyclin B1 in the same patients. Different grades of gastric cancer tissues were selected. Low, moderate and high indicate the expression levels of Cyclin B1 and CREPT. Scale bars, 100 μm.

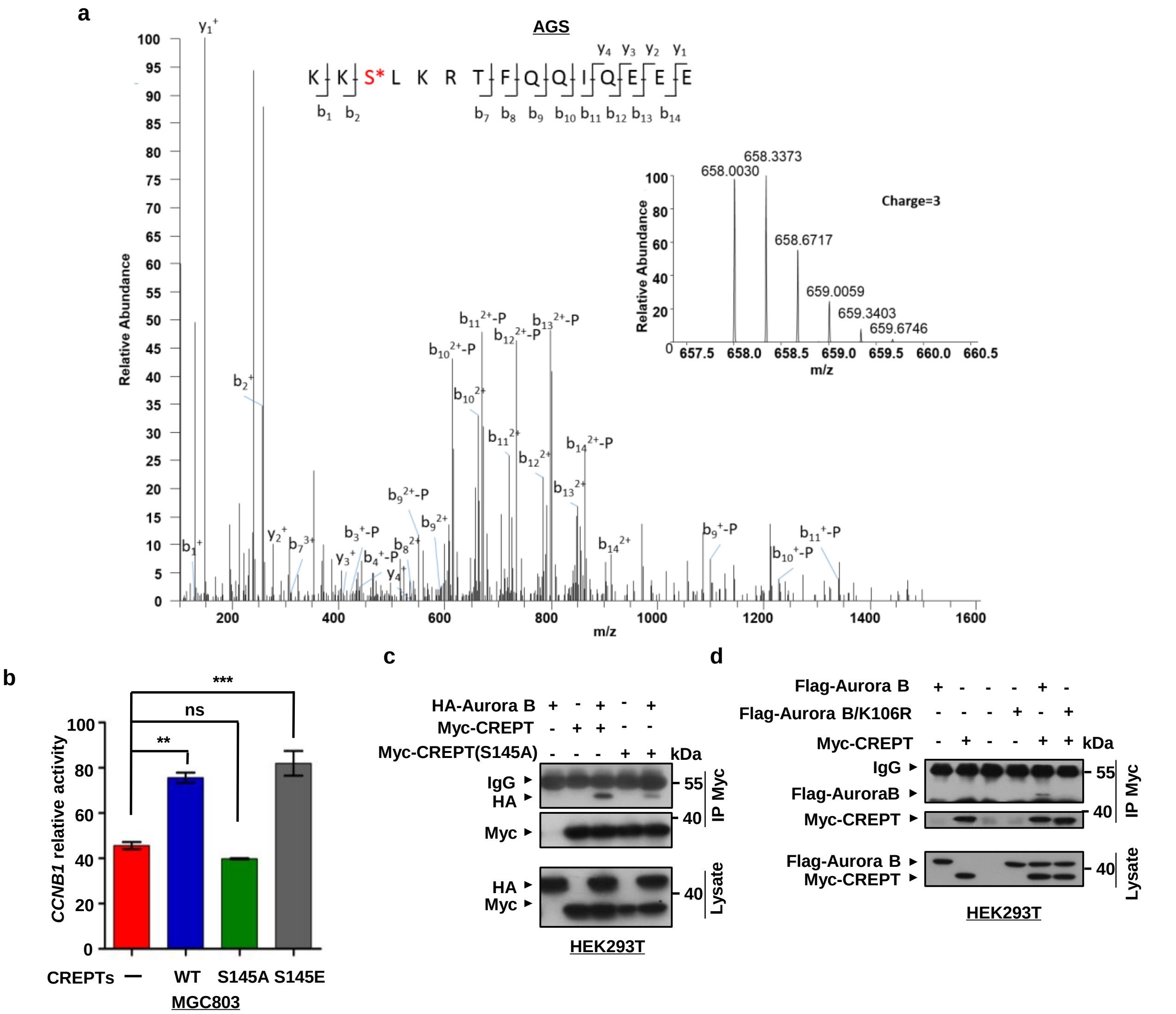

Figure S6. S145 is identified phosphorylated and is crucial for the transcription enhancement on *CCNB1* by an interaction with Aurora B.

(**a**) A mass spectrum (MS) analysis on the phosphorylation of CREPT. MS spectra of the peptide of KKpSLKRTFQQIQEEE is shown. pS indicates a phosphoSer residue, corresponding to S145. The Flag-tagged CREPT protein was immunoprecipitated from AGS cells, separated on SDS-PAGE, stained with Coomassie, and analyzed with Thermo Orbitrap Fusion Lumos mass spectrometer. The data were then processed by Proteome Discoverer (Version 1.4) software. (**b**) Phosphorylation of S145 is critical for the transcriptional activity of *CCNB1*. Note, S145A is a mutant lost the phosphorylation feature and S145E is a mutant that remains the phosphorylation feature. The experiments were performed in three independent repeats. **, p<0.01, ***, p<0.001. ns, no significant. (**c**) An inactive CREPT mutant shows a weak interaction with Aurora B. Myc-tagged wild type and S145A mutant (Myc-CREPT(S145A)) were co-transfected with HA-Aurora B into HEK293T cells. Immunoprecipiatation (IP) experiments were performed using an antibody against Myc from the cell lysates harvested from the cells after 24 h transfection. Western blots were performed for the complex and the lysate using indicated antibodies. (**d**) Wild type (WT) Aurora B, but not Aurora B(K106R) mutant, efficiently interacts with CREPT/RPRD1B. Myc-CREPT were co-transfected with HA-Aurora B or HA-Aurora B(K106R) into HEK293T cells. IP experiments were performed using an antibody against Myc from the cell lysates harvested from the cells after 24 h transfection.

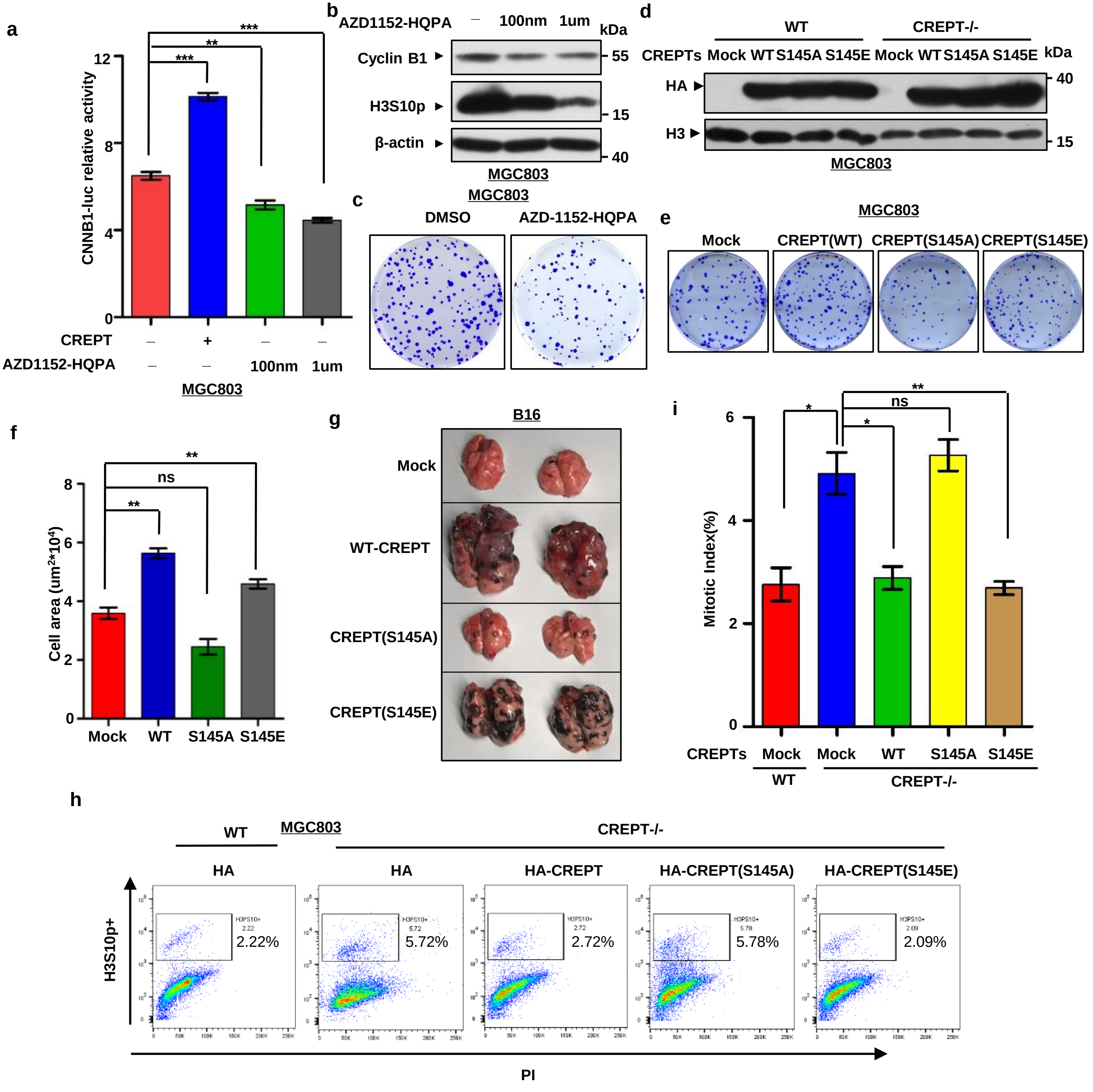

Figure S7. The figure legend is in the next page.

Figure S7. Inhibiting Aurora B impairs the *CCNB1* transcriptional activity and S145 phosphorylation is essential for CREPT in colony formation, tumor metastasis and mitosis.

(**a**) *CCNB1* transcriptional activity is suppressed by Aurora B inhibitors. *CCNB1* luciferase reporter and pRL-TK plasmids were co-transfected into MGC803 cells which were treated with DMSO and Aurora B inhibitor AZD1152-hydroxyquinazoline pyrazole anilide (AZD1152-HQPA). **, p<0.01. ***, p<0.001. (**b**) The expression of Cyclin B1 is suppressed when cells were treated with Aurora B inhibitor. Cells were treated with 100nM and 1 mM AZD1152-HQPA for 2 h and harvested by adding SDS loading buffer. (**c**) The colony formation is impaired when cells were treated with Aurora B inhibitor. 1000 cells were seeded and medium containing DMSO or AZD1152-HQPA was changed every 3 days. 10 days later, cells were harvested and stained with crystal violet. (**d**) Stable cell lines for overexpression CREPT mutants in CREPT wild type and deletion cells. MGC803 stable cell lines were generated with lentivirus infection. The expression of wild type (WT) CREPT, CREPT (S145A) and CREPT (S145E) was examined by a Western blot using an antibody against HA tag. H3 was used as the loading control. (**e**) Phosphorylation of CREPT at S145 is critical for the colony formation. Colony formation experiments were performed using cells with overexpression of CREPT and its mutants. 1000 cells per well were seeded and stained with crystal violet after 10 days. **(f**) A quantitative presentation of three independent experiments. Colony formation ability was expressed as cell area. The results are presented as mean +/- SD. **, p<0.01, ns, no significant. (**g**) CREPT(S145A), an inactive mutant, lost the ability to promote metastasis of B16 cells in vivo. CREPT and its mutants overexpressed B16 stable cells and its control cells were injected into the tail vein of 6-month-old C57/6J mice (n=5). After 21 days, mice were sacrificed and representative metastatic nodules on lungs were shown.(**h**) Wild type CREPT, but not the S145A mutant, rescues the mitotic index in CREPT/RPRD1B deletion cells. HA-vector and HA-CREPT/RPRD1Bs were exogenously expressed in CREPT deleted MGC 803 cells. Cells were harvested and stained with PI and H3S10p antibody. FACS analyses showed the H3S10p positive population which indicates the mitotic index. (**i**) A quantitative presentation of the mitotic index from three independent experiments. *p<0.05, **p<0.01.

Table S1. Primers used in this paper.

Primer name	Sequence	Description
si-CREPT #1	5’-GCAAGAACGAAGUGUUAUTT-3’	Used to deplete the expression of CREPT
si-CREPT #2	5’-GUCUGUUACUAGCAGAAUATT-3’	
non-target control	5’-UUCUCCGAAGUCACGUTT-3’	Used as a control siRNA
CREPT-KO1-F	5’-cacc G CGGTGCCACACGGAGACGAT-3’	Used to construct the CREPT deletion plasmid in CRISPR-Cas9 system
CREPT-KO1-R	5’-aaac ATCGTCTCCGTGTGGCACCG C-3’	
CREPT-KO2-F	5’-cacc G GCTAAGCCCCCTGTGACGTT-3’	
CREPT-KO2-R	5’-aaac AACGTCACAGGGGGCTTAGC C-3’	
S128A F	5’-CAGCTGAAGCTGGCCATGGAGGACTCCAAG-3’	Used to generate the CREPT mutants
S128A R	5’-TCCTCCATGGCC AGCTTCAGCTGCTGTATGAACT-3’	
S145A F	5’-CAGAAGAGAAGAAAGCCCTGAAA CGAAC-3’	
S145A R	5’-GGCTTTCTTCTCTTCTGTTGCTTTGG-3’	
T218A F	5’-ATTGGAAAAAATAGCCGACAAAGAGGCAGCTG-3’	
T218A R	5’-CTTTGTCGGCTATTTTTTCCAATAGAGAA-3’	
S227A F	5’-CAGCTGAACGTCTTGCCAAAACA GTAGATGA-3’	
S227A R	5’-TGCTTCATCTACTGTTTTGGCAAGACGTTC-3’	
S145E F	5’-CAGAAGAGAAGAAAGAGCTGAAACGAAC-3’	
S145E R	5’-CTCTTTCTTCTCTTCTGTTGCTTTGG-3’	
CCNB1-promoter-F	5’-ATTGGTACCCCGTGACTTCCAGCGCCAGGAGTCTCTATT-3’	Used to amplify the *CCNB1* promoter
CCNB1-promoter-R	5’- AATCTCGAGGCCATGGCTTCCTCTTCACCAGGCAGCAGCT-3’	
Cyclin B1 RT-F	5’-TATGCAGCACCTGGCTAAGA-3’	Used to test the mRNA level of Cyclin B1 with quantitative PCR assay
Cyclin B1 RT-R	5’-AGTGCAGAA TTCAGCTGTGG-3’	

Table S2. A correlation of CREPT expression and gastric tumor stages.

		CREPT Expression			Sum	%	r	p(two tail)
		Low	Moderate	High				
Gastric Cancer Stage	I	39	39	114	192	53.8	0.256**	p<0.01
	II	18	20	55	93	26.1		
	III	3	1	68	72	20.1		
Sum		60	60	237	357	100		
%		16.8	16.8	66.4	100			

r =Spearman correlation coefficient.

** Indicates that the correlation is significant at the 0.01 level (two tails).

Table S3. A correlation of CREPT expression and Cyclin B1 expression in gastric cancer tissues.

		CREPT Expression			Sum	%	r	p(two tail)
		Low	Moderate	High				
Cyclin B1 Expression	Low	125	59	8	192	53.8	0.172**	p<0.01
	Moderate	60	25	8	93	26.0		
	High	34	17	21	72	20.2		
Sum		219	101	37	357	100		
%		61.3	28.3	10.4	100			

r =Spearman correlation coefficient.

** Indicates that the correlation is significant at the 0.01 level (two tails).
